# Interventions for COVID-19 Vaccine Hesitancy: A Systematic Review and Narrative Synthesis

**DOI:** 10.3390/ijerph20126082

**Published:** 2023-06-08

**Authors:** Rowan Terrell, Abdallah Alami, Daniel Krewski

**Affiliations:** 1 School of Epidemiology and Public Health, University of Ottawa, Ottawa, ON K1S 5B6, Canada; 2 Risk Sciences International, Ottawa, ON K1P 5J6, Canada

**Keywords:** COVID-19 vaccines, vaccine hesitancy, vaccine refusal, systematic review, narrative synthesis, vaccine uptake, behavioral interventions, nudges

## Abstract

Vaccines effectively protect against COVID-19, but vaccine hesitancy and refusal hinder vaccination rates. This systematic review aimed to (1) review and describe current interventions for addressing COVID-19 vaccine hesitancy/refusal and (2) assess whether these interventions are effective for increasing vaccine uptake. The protocol was registered prospectively on PROSPERO and comprehensive search included Medline, Embase, CINAHL, PsycInfo, and Web of Science databases. Only studies that evaluated the effectiveness of non-financial interventions to address COVID-19 vaccine hesitancy were included, while those focusing intentions or financial incentive were excluded. Risk of bias for all included studies was evaluated using Cochrane risk of bias tools. In total, six articles were included in the review (total participants *n* = 200,720). A narrative synthesis was performed due to the absence of common quantitative metrics. Except for one randomized controlled trial, all studies reported that interventions were effective, increasing COVID-19 vaccination rates. However, non-randomized studies were subject to confounding biases. Evidence on the effectiveness of COVID-19 vaccine hesitancy interventions remains limited and further evidence is needed for the development of clear guidance on effective interventions to increase vaccine uptake.

## 1. Introduction

Vaccines are generally regarded as one of the greatest achievements in public health, having contributed significantly to declines in communicable disease morbidity and mortality [1]. Although vaccination coverage for most vaccine-preventable illnesses (e.g., measles, diphtheria, tetanus, and pertussis) is generally high among higher-income countries, significant gaps remain [2]. Importantly, under-vaccinated groups of the population tend to cluster at a local geographic level, resulting in pockets of the population that are vulnerable to outbreaks of vaccine-preventable illnesses [3].

The success of immunization programs is limited by rates of vaccine acceptance and uptake by the public. Vaccine hesitancy, a term coined by the Strategic Advisory Group of Experts on Immunization (SAGE) of the World Health Organization (WHO), refers to a delay in accepting vaccinations despite available and accessible vaccination services [4]. Increasing rates of vaccine hesitancy and refusal are a major public health concern: in fact, the WHO characterizes vaccine hesitancy as one of the top 10 threats to global health [5]. Consequently, elucidating the causes and determinants of vaccine hesitancy, as well as designing and evaluating potential strategies to address vaccine hesitancy, have emerged as increasingly important areas of public health research.

Individuals and groups may delay or refuse vaccination due to a variety of reasons, including reduced vaccine availability or accessibility [6], poor health literacy [7], scheduling difficulties or inconveniences [6], fear of adverse effects [8,9], mistrust of health authorities or healthcare providers [10,11], and personal spiritual or religious beliefs [12]. The modern anti-vaccination movement also plays a significant role in promoting vaccine hesitancy and resistance. Although opposition to vaccines is not a new phenomenon, the anti-vaccination movement has recently broadened its scope and scale at an alarming rate, in part due to the rise of social media platforms [13]. Misinformation, myths, and conspiracy theories about vaccine safety spread further and more rapidly on social media platforms when compared to verified vaccine information from reliable sources [14,15]. Research has also demonstrated that individuals who are vaccine-hesitant are more likely to have heard or read negative information about vaccines, most often receiving their vaccine information from internet sources [16,17,18].

When COVID-19 was declared a pandemic by the WHO in March 2020, vaccines were rapidly developed as a crucial preventative approach to reduce the morbidity and mortality of this highly contagious disease [19,20]. National COVID-19 immunization programs were launched across the world, starting in December 2020, with varying degrees of success. As of 6 November 2022, 83.1% of Canadians have received at least one dose of a COVID-19 vaccine [21]. Although vaccines are the most effective means of protecting individuals and communities from COVID-19, many remain hesitant or unwilling to receive a COVID-19 vaccine [22,23,24]. Certain groups are more likely to refuse COVID-19 vaccination, including people of colour [25], Indigenous people [26], politically conservative individuals [25], individuals without any post-secondary education [26], individuals with lower socio-economic status [26], and individuals who do not feel concerned about COVID-19 [26]. Understanding and addressing these demographic and socio-political factors that influence individuals’ attitudes on COVID-19 vaccines can support the development of successful interventions and strategies for addressing COVID-19 vaccine hesitancy.

Vaccine hesitancy is a complex, multi-faceted issue that can be difficult to address, both at the individual and at the population level. Current evidence regarding best practices and strategies to increase vaccine acceptance suggests that dialogue-based and multicomponent interventions are the most effective, although further research is needed [27,28]. A systematic review and meta-analysis by Vujovich-Dunn et al. (2021) that evaluated the effectiveness of decision aids (pamphlets, videos, web-based tools) found that some studies indicate that decision aids can reduce conflict in clinical vaccine decision making, increase vaccine intentions, and possibly increase vaccine uptake [29]. The authors noted that, although decision aids may be useful for promoting vaccine uptake, further research is needed to determine which elements and formats of these aids are most useful for mitigating vaccine hesitancy [29]. Similarly, a systematic review by Renosa et al. (2021) evaluated whether nudging interventions are effective at increasing vaccine uptake [30]. These nudging interventions—including reminders, incentives, and changes in vaccine decision making defaults (e.g., opt-out scheduling options)—were generally effective at increasing vaccine intention and uptake, although their specific impacts varied depending on context and setting. Finally, a 2015 review of published reviews and meta-analyses conducted by the SAGE working group concluded that there was no strong evidence to warrant a specific vaccine hesitancy intervention recommendation, indicating the need for further investigation [4].

One recent systematic review by Batteux and colleagues (2022) [31] evaluated the effectiveness of interventions for increasing COVID-19 vaccine uptake. This review captured primary research evaluating interventions for COVID-19 vaccine hesitancy and reported on the following outcomes: COVID-19 vaccine intentions and vaccination rates. Although the authors identified 39 studies in their review, the majority of the included studies measured vaccination intention outcomes as opposed to vaccination behaviour outcomes [31]. Additionally, the search strategy was not comprehensive as some key bibliographic databases were not searched, and it was conducted in July 2021, thus not capturing the most recent literature. Batteux et al. (2022) described several studies that evaluated whether certain interventions could increase COVID-19 vaccine intentions. Interventions that communicated (1) the benefits and safety of vaccination against COVID-19, (2) the importance of herd immunity against COVID-19, and (3) that others also intend to get vaccinated were all found to be associated with increases in COVID-19 vaccine intentions [31]. Text message reminders and educational videos were also found to be effective at increasing vaccine intentions in some contexts. Although this growing body of literature shows promise, these studies did not report behavioural outcomes (i.e., observed COVID-19 vaccination rates). Improvements in vaccine intentions or attitudes do not always translate to real-world behavioural changes [31]. We conducted a systematic review to determine which interventions described in the current literature are effective in changing COVID-19 vaccine behaviours. The objectives of this systematic review were to (1) review and describe current non-financial interventions for addressing COVID-19 vaccine hesitancy/refusal and (2) assess whether these interventions are effective at increasing vaccine uptake.

## 2. Materials and Methods

This review was conducted in accordance with the Preferred Reporting Items for Systematic Review and Meta-Analysis (PRIMSA) statement (Tables S1 and S2) [32]. The protocol for this systematic review was registered prospectively on PROSPERO, International prospective register of systematic reviews, registration number CRD42022343173.

### 2.1. Eligibility Criteria

Studies that evaluated the effectiveness of interventions to increase COVID-19 vaccine uptake among vaccine-hesitant adults were eligible for inclusion in the review. Studies focusing solely on interventions to increase vaccine uptake among pediatric populations were not included; however, studies that encompassed both adult and pediatric populations were included, but only results for adults were included in our narrative synthesis. Articles that evaluated the effect of financial incentives (e.g., lotteries) on vaccine uptake were considered beyond the scope of this review as these policies are more likely to financially benefit vaccine-accepting individuals. Studies that described or assessed factors associated with COVID-19 vaccine acceptance but did not evaluate any interventions were excluded as the objective of this review was to identify effective interventions. Outcomes of interest included any measures of COVID-19 vaccine uptake, such as through self-reports, or immunization/medical records. Studies that measured changes in vaccine intentions or attitudes were not considered eligible. Only studies published in English from 2020 to 5 July 2022 were considered as COVID-19 vaccines were unavailable prior to 2020. Review articles, editorials/correspondences, and abstracts with insufficient data were excluded. The detailed eligibility criteria for this review, including population, intervention, comparator, outcome, and study design, are summarized in Table 1.

### 2.2. Search Strategy

A comprehensive electronic search was conducted on 5 July 2022 using controlled vocabulary on the following bibliographic databases: Medline, Embase, PsycInfo, CINAHL, and Web of Science. The search strategy was developed and modified in consultation with a research librarian. Search terms included terms related to vaccination hesitancy, vaccination refusal, COVID-19, and COVID-19 vaccines. The search strategy was deliberately broad in order to capture any primary studies on interventions to increase COVID-19 vaccine uptake. The detailed systematic database search strategy is provided in Supplementary Tables S3–S7. The search strategy was complemented by screening all citations in the reference lists of three relevant and recent review articles [29,30,31]. Finally, a keyword internet search (“COVID-19 vaccine hesitancy intervention”) and scan of the first 1000 Google search results was conducted on 25 October 2022 to retrieve any additional web-based literature.

### 2.3. Study Selection

Screening of potentially eligible studies retrieved from the database search was performed using Covidence [33], an online systematic review software tool. Study selection was performed in duplicate using standardized screening questions. In Level 1 screening, 2 reviewers independently screened titles and abstracts of all citations retrieved using our search strategy. Any article considered as potentially relevant by either reviewer was retained for further screening. In Level 2 screening, full texts of relevant articles were independently assessed by both reviewers to determine eligibility for the review. Any discrepancies between reviewers were resolved through discussion. Any potentially relevant citations identified through the complementary searches proceeded to full-text review to determine their eligibility.

### 2.4. Data Extraction

The following data were extracted from included studies, using standardized forms: author, year of publication, study design, study participant characteristics, country of study, time of data collection, type and description of COVID-19 vaccine hesitancy intervention, comparator description, outcome measure for COVID-19 vaccine uptake, and effectiveness of the intervention. Data extraction was performed independently by 1 reviewer and was assessed by a second reviewer to ensure accuracy and completeness. Any discrepancies regarding data extraction were resolved through consensus discussion.

### 2.5. Risk of Bias Assessment

Risk of bias for all included studies was systematically evaluated using Version 2 of the Cochrane Risk of Bias tool (RoB 2) and the Cochrane Risk of Bias in Non-randomized Studies—of Interventions (ROBINS-I) depending on study type. Randomized controlled trials (RCTs) were evaluated using the RoB 2 tool, where a judgement of low, some concerns, or high risk of bias was assigned to each individual domain and to the study overall. Non-randomized studies were evaluated using the ROBINS-I tool, where a judgment of low, moderate, serious, critical, or unclear risk of bias was assigned to each domain. The risk of bias tools were used as a guide, and any additional insights beyond those provided by the tools directly were described in narrative form in the manuscript. Risk of bias assessment was performed independently by 1 reviewer and assessed by a second reviewer, with any discrepancies resolved by consensus.

### 2.6. Data Synthesis

Due to the variability in COVID-19 vaccine hesitancy interventions evaluated in the included studies, we did not conduct a meta-analysis to compute summary statistics or effect size estimates. Instead, a narrative data synthesis was undertaken, including presentation of study characteristics, methodologies, descriptions of the vaccine hesitancy/refusal interventions, and study findings in summary tables and in the main text. Study findings were interpreted while considering study population, context, and methodological quality. A narrative description of the included studies’ strengths and limitations was also included, along with answers to risk of bias tool items to provide perspective on the general quality of the literature (Tables S9 and S10).

## 3. Results

**Figure 1:**
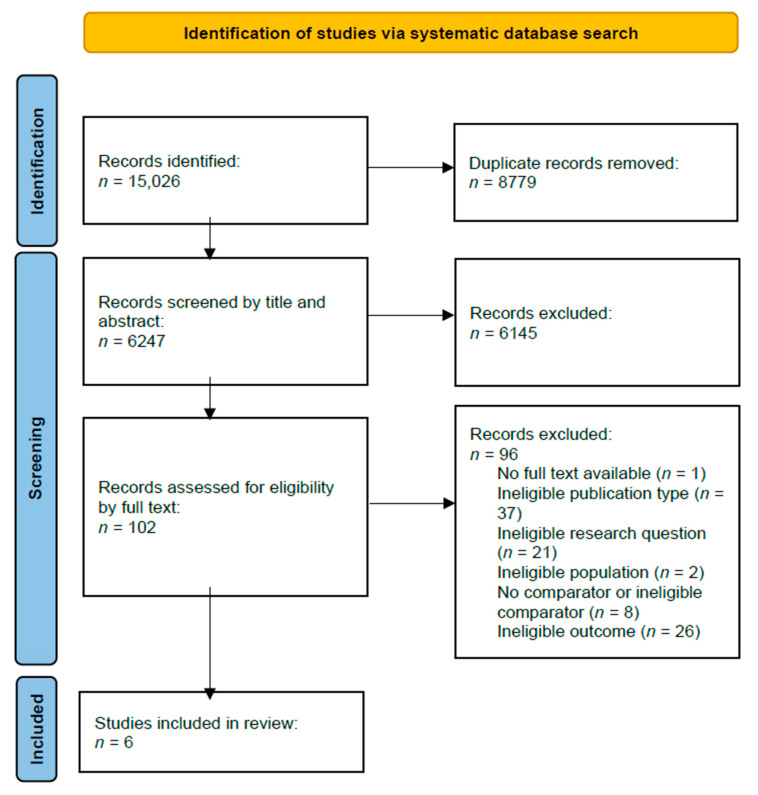
Systematic review flow diagram.

The study selection process is detailed in the PRISMA flow diagram included in Figure 1 [34]. A total of fifteen-thousand-twenty-six citations were retrieved from the five bibliographic databases in our systematic search strategy. Following automatic duplicate removal by Covidence, 6247 citations were retained for Level 1 (title and abstract) screening. A total of one-hundred-two potentially relevant articles identified in the systematic search progressed to Level 2 (full text) screening, of which six articles were selected for data extraction and risk of bias assessment. Two potentially relevant articles were identified from the complementary search of citations in the reference lists of three key review articles [29,30,31]; however, both were excluded after full-text review due to ineligible outcomes. Finally, in a complementary search of web-based literature, no potentially relevant citations were identified within the first 1000 Google search results. Articles that were excluded during full-text review are summarized in Table S8, along with reasons for their omission.

### 3.1. Overview of Included Studies

A summary of the characteristics and key findings of each included study is presented in Table 2. We identified a total of seven distinct interventions aimed at addressing COVID-19 vaccine hesitancy across the six publications included in our study. These interventions were evaluated using a range of methods, including five RCTs (with one article reporting on two sequential RCTs), one non-randomized controlled cluster trial, and one non-randomized uncontrolled before–after study. All but one of the studies included in the review took place in jurisdictions across the United States, with the remaining study conducted in Trento Province, Italy. In adherence with the eligibility criteria, all studies included adult participants who were COVID-19-vaccine-hesitant; however, one study [35] also included child participants 12 years of age or older. Sample sizes in the included studies were generally large but varied widely from a low of 2000 to over 90,000 participants. Several types of interventions to address COVID-19 vaccine hesitancy were discussed and evaluated in the six articles included in this review, including text messages/letters, culturally tailored outreach, infographics, informational videos, opt-out appointment booking systems, and multi-modal, complex interventions.

With the exception of the study by Crutcher and Seidler [35], all the included studies reported vaccination rates obtained through state/provincial or healthcare immunization records as the primary outcome. Crutcher and Seidler reported completion of a second COVID-19 vaccine dose as the outcome of interest, which was determined by the number of second-dose vaccine appointments per day in the vaccination site records. All included RCTs had a comparator group—generally a control group—with participants receiving standard care. The non-randomized controlled cluster trial reported the number of second vaccine dose recipients at the experimental site compared to other vaccine sites in the area; additionally, the authors compared the number of second vaccine dose recipients before and after the intervention was implemented. Similarly, the non-randomized uncontrolled before–after study conducted by Chan et al., 2022 [36] compared rates of complete vaccination among participants before and after the intervention was implemented.

### 3.2. Risk of Bias Assessment

The RCTs included in this review were evaluated using the Cochrane RoB 2 tool, in which a judgement of low concern, some concerns, or high risk of bias was assigned to each individual domain and to the study overall. The non-randomized interventions included in this review were evaluated using the Cochrane ROBINS-I tool, where a judgment of low, moderate, serious, critical, or unclear risk of bias was assigned to each ‘signaling question’ (questions that guide the risk of bias assessment process) to each domain and to the study overall. The overall risk of bias for each study is provided in Table 2; however, a more detailed summary of the risk of bias assessments for each study can be found in the Supplementary Materials in Tables S9 and S10. In general, the quality of the included studies was good. For the RCTs, three were rated as low for overall risk of bias, and one was considered to demonstrate some concerns. Although one article included in the review [37] reported on two sequential RCTs, only a single risk of bias assessment was performed for the article as the two RCTs were nearly identical methodologically, with the most recent study simply evaluating a second text-based reminder intervention on participants who remained unvaccinated. Common minor methodological concerns with the RCTs included lack of details provided on missing outcome data and on allocation concealment and blinding processes. Common methodological strengths among the RCTs included the presence of control/comparator groups, randomization processes that resulted in intervention/control groups with similar baseline characteristics, appropriate outcome assessment and statistical analysis plans that were established a priori, and blinding for outcome assessors and/or individuals administering the COVID-19 vaccine to participants.

Both the non-randomized studies were given a moderate overall risk of bias rating. The primary reasons for the moderate risk of bias ratings for the non-randomized studies were a lack of consideration of potentially confounding variables and that outcome assessors were aware of the intervention received by the participants. All the included studies benefitted from reporting an objective outcome measure (specifically, immunization records obtained from states/provinces or healthcare institutions) as opposed to subjective outcomes, which could have introduced bias.

### 3.3. Effectiveness of COVID-19 Vaccine Hesitancy Interventions

All the studies included in this review evaluated the effectiveness of different COVID-19 vaccine hesitancy interventions in increasing vaccination rates. Of the six included articles, five reported that the intervention was effective, resulting in an increase in vaccination rates when compared with pre-intervention rates and/or control groups. All but one of the included RCTs found that COVID-19 vaccine hesitancy interventions were effective in increasing vaccine uptake [37,38,40]. The authors of both non-randomized studies included in the review [35,36] concluded that their COVID-19 vaccine hesitancy interventions were effective; however, as the authors of these studies did not consider and control for potential confounders, these findings must be interpreted with caution.

#### 3.3.1. Text-Based Interventions

Studies testing whether text-based interventions reduce COVID-19 vaccine hesitancy yielded mixed findings. The format, delivery, and content of the interventions evaluated in these studies also varied. Two articles reported higher vaccine uptake among study participants who received COVID-19 vaccine appointment reminders/outreach [37,38]; however, one article reported no detectable increase in vaccination rates from a similar intervention [39].

Two sequential RCTs conducted by Dai and colleagues (2021) tested the effect of SMS-text-message-based reminders delivered to participants 1 day (first RCT) and 8 days (second RCT) after receiving a notification that they were eligible for the COVID-19 vaccine [37]. Participants enrolled in the first RCT (*n* = 93,354) were randomized into the following arms: holdout (control group), basic reminder, ‘ownership’ reminder, basic reminder with video, or ‘ownership’ reminder with video. The basic message reminded the participants to make a vaccine appointment online and provided a link to do so, while the ‘ownership’ reminder aimed to emphasize feelings of psychological ownership, stating “a COVID-19 vaccine has just been made available to you… Claim your dose today by making a vaccination appointment” (Dai et al., 2021 [37], p. 410). Additionally, a short video was paired with a reminder message in two of the intervention sub-arms. The 2 min video provided statistics on COVID-19 infections and transmission and presented the vaccines as a safe and effective solution. Participants randomized to any reminder type had higher vaccination rates when compared to vaccination rates in the control group. Text reminders resulted in a 25.7% relative increase in vaccine uptake over the following 4 weeks. The most effective reminder type was the reminder with ownership language, which yielded a 29.6% relative increase compared to the control group. The authors did not observe higher vaccine uptake among participants who received the informational video compared to the reminder-only participants.

Participants enrolled in the second RCT (*n* = 67,092) by Dai et al. (2021) [37] were randomized into the control group or received a second reminder that employed different behavioural messaging techniques to encourage COVID-19 vaccine uptake. The authors used a nested 2 × 3 factorial design in creating the content of the second reminders. The first factor emphasized either the personal benefits of receiving a COVID-19 vaccine as mentioned by Dai et al., 2021 (p. 411)—“to protect yourself, make your COVID-19 vaccine appointment here today”, or the societal benefits of getting vaccinated against COVID-19—“to protect your family, friends, and community, make your COVID-19 vaccine appointment here today” [37]. The second factor highlighted either the exclusivity of being able to receive the vaccine (early access framing) or framing getting vaccinated as facilitating a new path forward (fresh start framing). To summarize, participants in the second reminder RCT were randomized into seven possible arms: holdout (control group), basic ‘self’ reminder, basic ‘prosocial’ reminder, early access ‘self’ reminder, early access ‘prosocial’ reminder, fresh start ‘self’ reminder, or fresh start ‘prosocial’ reminder. Receiving a second reminder increased vaccine uptake by 17.2% relative to the control group, with all reminder types yielding a benefit. The authors also noted that, although the absolute increase in vaccination rates for the second reminder cohort was small, the effects are still noteworthy as the participants enrolled in this second RCT were likely more hesitant (as they remained unvaccinated after the first reminder RCT).

The RCT conducted by Lieu et al. (2022) [38] evaluated the effectiveness of mail- and electronic-secure-message-based outreach in increasing vaccine uptake among Black and Latino older adults. The authors specifically selected service areas that had lower vaccination rates and higher proportions of Black and Latino individuals. The participants (*n* = 8287) were randomized into the following arms: standard care (control group), standard outreach from their primary care physician via mail and/or electronic secure messaging, or similar outreach but with culturally tailored content. There were two rounds of outreach spaced 4 weeks apart. Study outreach 1 was an electronic secure message sent via an electronic health record portal, and study outreach 2 consisted of a postcard with similar content. Both outreach messages were informed by behavioural science, with message content including information about the safety and effectiveness of the COVID-19 vaccines and how to book a vaccine appointment. Culturally tailored outreach messages included this basic content, but also included additional factors considered to be relevant to the Black and Latino ethnic groups. As outlined by Lieu et al., 2022 (pp. 3–4), these factors included cost—“The vaccine is available at no cost”, immigration status—“The vaccine is available […] regardless of your immigration status. We want to reassure you that we never share your personal information with outside agencies”, and ethnic disparities observed during the COVID-19 pandemic—“Getting vaccinated protects those who have been most harmed by COVID-19 like Latinos/Latinx, Black/African Americans, and many others” [38]. Participants were followed for 8 weeks after the initial outreach to determine if they received a dose of a COVID-19 vaccine. Although there was no statistically significant difference in vaccination rates between the standard and culturally tailored outreach groups, both outreach strategies resulted in higher vaccination rates when compared to the control group after 8 weeks of follow up (adjusted HR: 1.17; 95% CI, 1.04–1.31 for the standard group and aHR: 1.22; 95% CI, 1.09–1.37 for the culturally tailored group). The authors stipulated that there was no particular benefit to the culturally tailored outreach because these messages had very little culturally specific content. They further hypothesized that more intensive culturally tailored outreach may be beneficial.

Another RCT included in this review, conducted by Mehta and colleagues (2022) [39], evaluated whether a text-message-based intervention would result in increased vaccine uptake among study participants. Participants (*n* = 16,045) were randomized to receive a telephone call from a call centre (this was considered as the ‘usual care’/control group), a text message with instructions to call a hotline, or a text message and an outbound telephone call if they responded. The trial conducted by Mehta et al., 2022 (p. 4) employed a factorial design: the participants in the intervention groups were further randomly assigned to four different types of messaging content informed by behavioural science principles, including clinician endorsement –“Dr. XXX recommends that you receive the vaccination”, endowment—“We have reserved a COVID-19 vaccine appointment for you”, scarcity—“You have been selected to receive from the limited supply of COVID-19 vaccine”, or a standard message—“Our records show you are eligible for your COVID-19 vaccine” [39]. The authors reported the percentage of study participants who completed the first dose of the COVID-19 vaccine within 1 month after receiving the intervention. Unlike the studies conducted by Dai et al. (2021) [37] and Lieu et al. (2022) [38], the authors of this trial reported no statistically significant difference in vaccine uptake between any intervention group (either standard and behaviourally informed message content) and the telephone call (control) group. Additionally, the authors found that there was no significant benefit from behaviourally informed messaging when compared to the standard text message. In their discussion, the authors noted that the study’s null findings may have been influenced by the study period: the trial took place between April and July 2021, at which point COVID-19 vaccines were already widely available and accessible.

#### 3.3.2. Infographics

One study included in this review evaluated whether an infographic that emphasizes the importance of returning for a second COVID-19 vaccine dose was effective in increasing rates of completion of a two-dose regimen [35]. Specifically, the infographic used pictures and analogies to describe how mRNA COVID-19 vaccines work, in lay terms, and showed, using a simple bar graph, that better protection from COVID-19 can be achieved by receiving a second dose. The intervention consisted of handing out the infographic sheet to individuals receiving their first vaccine dose at the vaccination site. The authors compared the number of second-dose vaccine recipients before the intervention to the number of second-dose recipients during the effect period (21 days after the infographic was distributed since participants were advised to return for their second dose 3 weeks after their first dose). They also compared the rate of second-dose appointments at the study site and county-wide second-dose vaccination rates. The authors reported that the study site served 15.8% more second-dose recipients when compared to all other county vaccine sites in the same timeframe. Additionally, the proportion of second-dose recipients at the study site in the effect period surpassed the proportion of second-dose recipients at the same site before the infographic was distributed. Although the authors concluded that infographics are useful tools that may increase vaccine uptake and/or completion of a two-dose vaccine regimen, these results must be interpreted with caution. The authors of this non-randomized study did not collect baseline demographic data from participants at the vaccination sites and did not consider potentially confounding variables that may have resulted in differing vaccination rates at other sites in the county. Furthermore, the authors did not describe that the increase in second-dose recipients at the study site may have been due to the uneven pace in vaccine rollout; perhaps more individuals returned for their second dose during the effect period because, as time went on, more and more individuals could access—or were newly eligible for—the COVID-19 vaccine.

#### 3.3.3. ‘Opt-Out’ Interventions

One RCT by Tentori et al. (2022) [40] evaluated whether a nudging strategy that leverages status quo bias could improve vaccine uptake among adults aged 50–59 years living in a region in Italy where vaccine hesitancy was high. The participants (*n* = 2000) in this trial were randomized to either receive a letter with instructions on how to book a COVID-19 vaccine appointment (‘opt-in’ group) or a letter with details (date, time, location) of a pre-booked COVID-19 vaccine appointment with instructions on how to modify the appointment, if desired (‘opt-out’ group). After a 19-day interval (the time between the participants receiving the letters and the last day of pre-booked appointments in the opt-out group), the authors compared the vaccination rates between the opt-in and opt-out intervention groups. The results from their logistic regression analysis indicated that the participants in the opt-out group had significantly higher COVID-19 vaccine uptake when compared to the opt-in group (odds ratio: 1.37, 95% CI, 1.032–1.809; *p* = 0.029). The authors concluded that a simple switch to a different default option may be a powerful tool in increasing vaccine acceptance, even among particularly reluctant populations.

## 4. Discussion

This systematic review aimed to capture and assess evidence on the effectiveness of COVID-19 vaccine hesitancy interventions. The studies included in this review tested a broad range of interventions to increase COVID-19 vaccine uptake, including reminders, nudging strategies, infographics, and multi-modal outreach. The COVID-19 vaccine hesitancy interventions evaluated in the included studies were found to be generally effective at increasing COVID-19 vaccination rates. The only included study that reported null findings was the RCT conducted by Mehta and colleagues (2022) [39]. However, the results of this RCT should be contextualized: COVID-19 vaccines were already widely available and accessible in the region prior to the beginning of the study period (April–July 2021), and participants had already received previous outreach from public health agencies. Both of these factors may have contributed to the null findings. On the other hand, the two sequential RCTs conducted by Dai et al. (2021) [37] and the RCT conducted by Lieu et al. (2022) [38]—which were conducted earlier (January–May 2021 and March–May 2021), when community demand for vaccines was higher—found that similar text-based interventions significantly increased COVID-19 vaccination rates. The trials conducted by Dai et al. (2021) [37] also had control groups that received no outreach (‘holdout’ arms), whereas the Mehta et al. (2022) [39] trial had an active comparator group. If the Mehta et al. (2022) [39] trial had included a true control or ‘holdout’ arm, an increase in vaccination rates may have been observed in the intervention groups.

External factors likely influenced the results of the two non-randomized studies included in this review. In particular, the study conducted by Crutcher and Seidler (2021) [35], was subject to high risk of bias in the confounding domain: the authors did not control for any potentially confounding variables, nor did they collect or analyze any demographic data for the participants presenting at the study’s immunization site. This likely biased the comparison to the county-wide rates of second COVID-19 vaccine dose completion and the pre-intervention and post-intervention comparisons in this study. Their primary outcome measure—second dose completion rate—at the study immunization site may have differed from the second dose completion rates in all of Los Angeles County due to intrinsic community demographics. Specifically, it is possible that the Lincoln Park immunization site (the study setting) may have attracted more visitors with lower levels of vaccine hesitancy compared to other immunization sites. Further, the pre–post comparison is subject to temporal confounding. An increase in visitors returning for their second dose during the study effect period (post-intervention) may have been influenced by the uneven pace of COVID-19 vaccine rollouts; perhaps there were more individuals returning for their second dose due to external factors, such as increased vaccine availability.

Many of the interventions assessed in this review included content related to vaccine safety and efficacy. However, delays in vaccine uptake may not always arise from concerns about the vaccines per se but can also originate from procrastination or scheduling difficulties [41]. The results from the Italian RCT conducted by Tentori et al. (2022) [40] suggest that COVID-19 vaccine hesitancy can be mitigated with strategies such as an opt-out vaccine appointment scheduling system. In their trial, Tentori et al. (2022) [40] demonstrated that this default-changing strategy may be an efficient and effective approach to increasing COVID-19 vaccine uptake. Similarly, a pre-print study by Serra-Garcia and Szech (2021) [42] demonstrated that this default change resulted in increased vaccine intentions among student participants during the early stages of the pandemic. Although preliminary evidence suggests that opt-out vaccine appointment scheduling systems may be effective at increasing COVID-19 vaccine uptake [40] and intentions [42], it is not clear how this default-changing strategy may interact with other interventions, such as those that address common vaccine concerns, such as safety and efficacy.

Rates of COVID-19 vaccine hesitancy are generally higher among visible minority groups when compared to the general population, particularly in the United States [43]. Two studies included in this review focused on increasing vaccine uptake among specific ethnic groups that are more likely to be vaccine-hesitant, namely Black, Latino, and multi-racial individuals living in the United States [36,38]. The non-randomized intervention study conducted by Chan et al. (2022) [36] described and evaluated a multi-modal COVID-19 vaccine hesitancy intervention among a cohort of healthcare employees, with a focus on Black, Hispanic, and multi-racial minorities. The multi-modal intervention strategy—which included town halls, conveniently located vaccination stations, and educational materials and counselling on vaccine safety and efficacy—was found to be most effective at increasing COVID-19 vaccine uptake among Black and Hispanic employees specifically [36]. Although the RCT conducted by Lieu et al. (2022) [38] revealed no statistically significant difference in vaccination rates between standard and culturally tailored outreach groups, outreach from primary care providers resulted in higher vaccination rates among the Black and Latino older adults. Interestingly, another experimental study reported that culturally tailored messaging was associated with increased vaccine intentions among Black Americans [43]. Further research is needed to determine whether culturally tailored messaging is an effective tool for increasing COVID-19 vaccine uptake. Although the participants in other studies included in this review were less ethnically and racially diverse, some authors completed subgroup analyses and discussed the effectiveness of COVID-19 vaccine hesitancy interventions among specific ethnic groups. In the first RCT conducted by Dai et al. (2021) [37], the authors recognized that the sample largely consisted of older Caucasian adults; however, the increases in vaccine appointments and vaccination rates were comparable across all included ethnic groups (Caucasian, Hispanic, Black, and Asian). The subgroup analyses from the Mehta et al. (2022) [39] RCT also indicated that there were no meaningful differences between ethnic groups in terms of vaccination rates in response to the outreach intervention.

Recent reviews have reported that nudges, decision aids, and other vaccine hesitancy interventions are likely effective at increasing vaccination rates [29,30,31]. A systematic review and meta-analysis by Vujovich-Dunn and colleagues (2021) identified five RCTs that evaluated decision aids for vaccine decision making [29,44,45,46,47,48]. The decision aids evaluated in the included studies consisted of web- and paper-based vaccine educational materials. The authors reported that these decision aids slightly increased vaccine uptake; however, when higher risk of bias studies were excluded from their meta-analysis, this increase was not apparent [29]. Additionally, the included RCTs that measured vaccine intentions (as opposed to vaccination rates) as an outcome reported that decision aids significantly increased vaccine intentions [29]. Renosa et al. (2021) conducted a systematic review to evaluate whether nudging interventions are effective at increasing vaccine uptake [30]. These nudging interventions included reminders, incentivizing vaccination (including financially), invoking social norms, and changing vaccine defaults (e.g., opt-out scheduling options) [30]. Renosa et al. (2021) concluded that nudging-based interventions may be effective at increasing vaccine intention and uptake, although their effectiveness varied depending on the population, context, and setting [30]. However, these two reviews did not specifically evaluate vaccine hesitancy interventions for COVID-19. The systematic review by Batteux et al. (2022) did evaluate the effectiveness of interventions to increase COVID-19 vaccine uptake, although their search strategy was less comprehensive and did not capture more recent literature (published after July 2021); their review also focused primarily on intervention studies that reported COVID-19 vaccine intention as the primary outcome [31]. Unlike our review, the systematic review by Batteux et al. (2022) included studies that evaluated the effectiveness of financial incentives [31]. Similar to our review, Batteux and colleagues found that interventions that communicated that COVID-19 vaccines are safe and effective and emphasized the benefits of getting vaccinated were generally successful at increasing vaccine uptake. Personalized text interventions and reminders, such as those highlighting that a vaccine dose has been made available to the individual, were also found to be effective at increasing vaccine uptake [31].

As previously mentioned, studies that evaluated the effectiveness of financial incentives were considered ineligible for inclusion since individuals who are already vaccine-accepting financially benefit from such policies. Financial interventions are generally referred to as “conditional cash lotteries” in the literature—providing an opportunity for financial gain only if a specific behaviour is adopted [49]. To date, most of the scientific evidence evaluating the impact of conditional cash lottery programs on COVID-19 vaccination rates originates from the United States [49] since several US jurisdictions implemented cash lotteries to incentivize vaccination. These programs have yielded mixed results in terms of increasing COVID-19 vaccination rates.

The “Vax-a-Million” cash lottery implemented in the state of Ohio serves as an interesting case study, with its impacts having been evaluated by several researchers [49,50,51,52,53]. In May 2021, the Ohio Department of Health announced the Vax-a-Million initiative: Ohioan adults (>18 years) who had received at least one dose of a COVID-19 vaccine were eligible to enter to win in a series of 5 weekly state-wide draws for USD 1 million [54]. Initial evaluations of the Ohio Vax-a-Million initiative indicated that it was effective at increasing COVID-19 vaccine uptake [49,52]. In contrast, other investigations concluded that vaccination rates did not increase in Ohio after the lottery was introduced [50,53]. This discordance was investigated by Mallow et al. (2022) [51], who sought to clarify the effectiveness of the Ohio Vax-a-Million lottery by analyzing state immunization data. Their analysis revealed that the lottery initiative was indeed accompanied with an increase in COVID-19 vaccination rates, particularly in lower-income counties. Similarly, a cross-sectional study conducted by Acharya and Dhakal (2021) [55] compared self-reported and state immunization data in 11 states that implemented a financial incentive program and in 28 states with no financial incentive programs in March–July 2021. According to their analyses (which included *n* = 403,714 individuals), these programs increased vaccine uptake nationally, but were not effective in some states (Arkansas, Kentucky, West Virginia). In the 2022 systematic review conducted by Batteux and colleagues, the authors noted that evidence on these interventions remains heterogeneous. Finally, while there is some evidence to suggest that offering financial incentives can increase COVID-19 vaccine uptake, some researchers warn that incentives, particularly small ones, can backfire and actually reduce vaccine intentions [42,56].

Vaccination is an important public health strategy for protecting individuals against COVID-19 hospitalization and death [20]. Determining which interventions are most effective at increasing uptake among vaccine-hesitant populations may inform future vaccine rollout strategies for COVID-19 and other infectious disease outbreaks. This systematic review demonstrates that a range of interventions have been tested to increase COVID-19 vaccine uptake, although the body of evidence is currently limited and further evidence is needed for the development of clear vaccine hesitancy guidance.

Our key findings on interventions for increasing COVID-19 vaccine uptake are summarized in Table 3. These observations are informed by the findings of articles included in this systematic review, findings from studies that evaluated effectiveness of similar interventions for other vaccine-preventable illnesses (e.g., influenza), and findings from studies that evaluated the effects of intervention on COVID-19 vaccination intentions. Policymakers and decision makers should be aware that there are currently very few large-scale RCTs that evaluate the impacts of vaccine hesitancy interventions on COVID-19 vaccination rates. However, this body of evidence is continually evolving, and future intervention studies may serve to clarify which interventions are most effective in increasing COVID-19 vaccine acceptance.

## 5. Strengths and Limitations

Systematic reviews provide a comprehensive and objective synthesis of available evidence, and help clinicians and policymakers identify effective interventions and improve health outcomes [59]. We conducted a systematic review with a comprehensive search strategy and rigorous screening process to address our research question. The comprehensive database search was designed in collaboration with a research librarian and captured a broad range of evidence on COVID-19 vaccine hesitancy. We excluded studies that reported COVID-19 vaccine intentions or attitudes as the primary outcome since these outcomes are not always predictive of actual vaccine behaviour (e.g., observed vaccination rates) [31]. This systematic review is subject to some limitations. First, since the interventions described and evaluated in the included articles were all unique, we were unable to conduct a meta-analysis due to high levels of heterogeneity and instead undertook a narrative synthesis of the available evidence. The articles included in this review all originated from the United States (*n* = 5) and Italy (*n* = 1), both of which are high-income countries. While the results from studies conducted in the US and Italy provide valuable insights, it is important to acknowledge that generalizability to other high-income countries may be limited due to potential differences in social, cultural, and health system contexts. Additionally, the relatively small number of studies (*n* = 6) meeting our strict inclusion criteria may have limited the breadth of interventions evaluated. The present review did not include studies that evaluated interventions for increasing vaccination rates for other vaccine-preventable diseases (e.g., influenza, measles); however, future reviews may consider collating, summarizing, and extrapolating the findings of these intervention studies for COVID-19 vaccine hesitancy specifically. Finally, research on the effectiveness of interventions for COVID-19 vaccine hesitancy has been initiated only during the current pandemic and is continually evolving. Our observations on the interventions investigated to date, although informed by the best available evidence, should be considered as preliminary and subject to updating as new information becomes available.

## 6. Conclusions

COVID-19 vaccine hesitancy is a complex, multi-faceted issue, and effective interventions to address this issue need to be developed and evaluated. Our systematic review indicates that certain non-financial interventions, notably appointment reminders and opt-out scheduling systems, appear to encourage vaccine uptake. Other approaches, such as multi-modal interventions and infographics, also show potential, although more research is required to ascertain their effectiveness and feasibility for diverse populations. As knowledge surrounding COVID-19 vaccine hesitancy evolves, more robust evidence-based guidance on future interventions to increase vaccine uptake may be developed.

## Figures and Tables

**Table 1:** Eligibility criteria.

**Research question**	Study implements and evaluates a non-financial intervention intended to address COVID-19 vaccine hesitancy and increase vaccine uptake
**Population**	Adults who are hesitant to receive the COVID-19 vaccine
**Intervention**	Intervention implemented to address COVID-19 vaccine hesitancy and increase COVID-19 vaccination rates
**Comparator**	Eligible comparators included: comparison between experimental group(s) vs. control group(s), comparison between >2 intervention groups (receiving different interventions), or comparison between COVID-19 vaccination rates before and after implementing the intervention
**Outcome**	COVID-19 vaccination rate (i.e., self-reported, immunization/medical records)
**Study design**	Peer-reviewed comparative studies (i.e., randomized controlled trials, non-randomized trials, cohort studies, case-control studies)

**Table 2:** Characteristics and key findings of included studies.

Citation	Study Design	Participants and Study Period	Intervention Type	Comparator	Outcome Variable	Key Findings	Risk of Bias
Chan et al., 2022 [36]	Non-randomized uncontrolled before–after study	Healthcare employees (*n* = 13,942) living in Oregon and Washington state, USA (August–October 2021)	Complex intervention with multiple components (including town halls, meetings, optional vaccine counselling, etc.)	Before–after comparison	Rate of complete vaccination among participants (immunization records obtained via state CDC Immunization Information Systems and reconciled with EMR employee data)	9.8% absolute increase in complete vaccination rate after intervention	Moderate
Crutcher & Seidler, 2021 [35]	Non-randomized controlled cluster trial	Adults and children >12 years (number of participants not reported) living in Los Angeles, California, USA (June–July 2021)	Educational infographic	Control group (county-wide) comparison and before–after comparison	Completion of second vaccine dose for COVID-19 (determined from Lincoln Park vaccination site records)	The Lincoln Park vaccination site served 15.8% more second-dose recipients when compared to all of Los Angeles County vaccine sites in the same timeframe	Moderate
Dai et al., 2021 [37]	2 sequential randomized controlled trials	Adult patients (*n* = 93,354 for first trial, *n* = 67,092 for second trial) from the UCLA Health patient list in Los Angeles, California, USA (January–May 2021)	Text-based reminders with or without video	Control group (for both trials)	Vaccination rates (immunization records available through the UCLA healthcare system)	The first reminder (first trial intervention) resulted in a 3.57% absolute increase in vaccine uptake, and the second reminder (second trial intervention) resulted in a 1.06% absolute increase.	Low
Lieu et al., 2022 [38]	Randomized controlled trial	Latino and Black adults (*n* = 8287) living in Northern California, USA (March–May 2021)	Culturally tailored outreach via letters and secure electronic messaging	Control group (usual care)	Completion of at least 1 dose of a COVID-19 vaccine (according to state immunization records)	Patients receiving both standard (adjusted HR: 1.17; 95% CI, 1.04–1.31) and culturally tailored (aHR: 1.22; 95% CI, 1.09–1.37) outreach demonstrated higher vaccination rates compared to usual care	Some concerns
Mehta et al., 2022 [39]	Randomized controlled trial	Adults (*n* = 16,045) living in Philadelphia, Pennsylvania, USA (April–July 2021)	Text message, with standard, scarcity, clinical endorsement, and endowment message framing	Control group (received standard telephone call)	Proportion of patients who completed the first dose of the COVID-19 vaccine within 1 month of intervention (according to EMR)	No detectable increase in vaccine uptake among patients receiving text messages or behaviourally informed message content compared to telephone calls only	Low
Tentori et al., 2022 [40]	Randomized controlled trial	Adults (*n* = 2000) living in Trento Province, Italy (July–August 2021)	Vaccine appointment booking, with option to ‘opt-out’	Control group (received usual ‘opt-in’ option to schedule vaccination appointment)	Vaccination rate (obtained from provincial records)	32% relative increase in vaccination rate among those in ‘opt-out’ group when compared to the ‘opt-in’ (control) group	Low

**Table 3:** Observations on interventions for increasing COVID-19 vaccination.

Intervention	Observation and Rationale
Text-based reminders and outreach	Basic reminders and outreach delivered by SMS text messages, letters, or secure electronic messaging may be effective at increasing COVID-19 vaccine uptake [37,38]. Text message reminders have similarly been shown to increase influenza vaccination rates [57]. However, this type of outreach may not always be effective, particularly if individuals have already received previous reminders [39].
Videos	Authors of 1 high-quality RCT did not observe higher vaccine uptake among participants who received an informational video compared to participants who only received a text reminder [37]. However, a study by Khatri et al. (2022) reported that an educational video on COVID-19 vaccines was associated with increased COVID-19 vaccine intentions [58].
Infographics	Further research is needed to determine whether educational infographics are an effective intervention for COVID-19 vaccine hesitancy. However, results from a moderate-quality non-randomized study suggest that infographics may encourage first vaccine dose recipients to return for their second dose [35].
Opt-out vaccine appointment scheduling	Preliminary evidence suggests that opt-out vaccine appointment scheduling systems may be effective at increasing COVID-19 vaccine uptake [40] and intentions [42].
Multi-modal interventions	Results from a moderate-quality non-randomized study suggest that a multi-modal intervention approach (with elements such as town halls, staff meetings, and vaccine safety education and counselling) may be effective at increasing vaccine uptake, specifically among healthcare workers [36]. Further research is needed to determine whether certain components of the multiple intervention approach described by Chan et al. (2022) are more effective than others [36], and whether this approach could be feasibly generalized to a broader population.

## Data Availability

The datasets analyzed for this study can be found in the manuscript and Supplementary Materials.

## Supplementary Materials

The following supporting information can be downloaded at: ‘https://www.mdpi.com/article/10.3390/ijerph20126082/s1, Table S1. PRISMA 2020 checklist, Table S2. PRISMA 2020 for abstracts checklist, Table S3. Medline search strategy, Table S4. Embase search strategy, Table S5. PsycInfo search strategy, Table S6. CINAHL search strategy, Table S7. Web of Science search strategy, Table S8. Articles excluded in full-text review and reasons for exclusion, Table S9. Risk of bias assessments for randomized intervention studies, Table S10. Risk of bias assessments for non-randomized intervention studies.
