# Supplementary material for: Interventions for COVID-19 Vaccine Hesitancy: A Systematic Review and Narrative Synthesis
Source: Int J Environ Res Public Health. 2023 Jun 8;20(12):6082. doi: 10.3390/ijerph20126082 (PMC10298220; doi:10.3390/ijerph20126082)
Supplement: Supplementary file 1 [file ijerph-20-06082-s001.zip › ijerph-2332612-SM.pdf]

Supplementary materials

Interventions for COVID-19 vaccine hesitancy: A systematic review and narrative synthesis

Rowan Terrell, Abdallah Alami, and Daniel Krewski

The supplementary material provides information on PRISMA reporting checklists (Tables S1-2), systematic search strategies (Tables S3-7), articles that were excluded in full-text review and reasons for their exclusion (Table S8), and details on the risk of bias assessments for included studies (Tables S9-10). A full list of the supplementary tables is provided below.

- Table S1. PRISMA 2020 checklist
- Table S2. PRISMA 2020 for abstracts checklist
- Table S3. Medline search strategy
- Table S4. Embase search strategy
- Table S5. PsycInfo search strategy
- Table S6. CINAHL search strategy
- Table S7. Web of Science search strategy
- Table S8. Articles excluded in full-text review and reasons for exclusion
- Table S9. Risk of bias assessments for randomized intervention studies
- Table S10. Risk of bias assessments for non-randomized intervention studies

Table S1. PRISMA 2020 checklist

Section and Topic	Item #	Checklist item	Location where item is reported
**TITLE**			
Title	1	Identify the report as a systematic review.	Page 1
**ABSTRACT**			
Abstract	2	See the PRISMA 2020 for Abstracts checklist.	Page 3
**INTRODUCTION**			
Rationale	3	Describe the rationale for the review in the context of existing knowledge.	Pages 4-7
Objectives	4	Provide an explicit statement of the objective(s) or question(s) the review addresses.	Page 7
**METHODS**			
Eligibility criteria	5	Specify the inclusion and exclusion criteria for the review and how studies were grouped for the syntheses.	Page 8
Information sources	6	Specify all databases, registers, websites, organisations, reference lists and other sources searched or consulted to identify studies. Specify the date when each source was last searched or consulted.	Pages 9-10
Search strategy	7	Present the full search strategies for all databases, registers and websites, including any filters and limits used.	Tables S3-7
Selection process	8	Specify the methods used to decide whether a study met the inclusion criteria of the review, including how many reviewers screened each record and each report retrieved, whether they worked independently, and if applicable, details of automation tools used in the process.	Page 10
Data collection process	9	Specify the methods used to collect data from reports, including how many reviewers collected data from each report, whether they worked independently, any processes for obtaining or confirming data from study investigators, and if applicable, details of automation tools used in the process.	Pages 10-11
Data items	10a	List and define all outcomes for which data were sought. Specify whether all results that were compatible with each outcome domain in each study were sought (e.g. for all measures, time points, analyses), and if not, the methods used to decide which results to collect.	Page 11
	10b	List and define all other variables for which data were sought (e.g. participant and intervention characteristics, funding sources). Describe any assumptions made about any missing or unclear information.	Pages 10-11
Study risk of bias assessment	11	Specify the methods used to assess risk of bias in the included studies, including details of the tool(s) used, how many reviewers assessed each study and whether they worked independently, and if applicable, details of automation tools used in the process.	Page 11
Effect measures	12	Specify for each outcome the effect measure(s) (e.g. risk ratio, mean difference) used in the synthesis or presentation of results.	N/A

Section and Topic	Item #	Checklist item	Location where item is reported
Synthesis methods	13a	Describe the processes used to decide which studies were eligible for each synthesis (e.g. tabulating the study intervention characteristics and comparing against the planned groups for each synthesis (item #5)).	N/A
	13b	Describe any methods required to prepare the data for presentation or synthesis, such as handling of missing summary statistics, or data conversions.	N/A
	13c	Describe any methods used to tabulate or visually display results of individual studies and syntheses.	N/A
	13d	Describe any methods used to synthesize results and provide a rationale for the choice(s). If meta-analysis was performed, describe the model(s), method(s) to identify the presence and extent of statistical heterogeneity, and software package(s) used.	N/A
	13e	Describe any methods used to explore possible causes of heterogeneity among study results (e.g. subgroup analysis, meta-regression).	N/A
	13f	Describe any sensitivity analyses conducted to assess robustness of the synthesized results.	N/A
Reporting bias assessment	14	Describe any methods used to assess risk of bias due to missing results in a synthesis (arising from reporting biases).	N/A
Certainty assessment	15	Describe any methods used to assess certainty (or confidence) in the body of evidence for an outcome.	N/A
**RESULTS**			
Study selection	16a	Describe the results of the search and selection process, from the number of records identified in the search to the number of studies included in the review, ideally using a flow diagram.	Figure 1
	16b	Cite studies that might appear to meet the inclusion criteria, but which were excluded, and explain why they were excluded.	Table S8
Study characteristics	17	Cite each included study and present its characteristics.	Table 2
Risk of bias in studies	18	Present assessments of risk of bias for each included study.	Tables S9-10
Results of individual studies	19	For all outcomes, present, for each study: (a) summary statistics for each group (where appropriate) and (b) an effect estimate and its precision (e.g. confidence/credible interval), ideally using structured tables or plots.	Table 2
Results of syntheses	20a	For each synthesis, briefly summarise the characteristics and risk of bias among contributing studies.	Pages 17-18
	20b	Present results of all statistical syntheses conducted. If meta-analysis was done, present for each the summary estimate and its precision (e.g. confidence/credible interval) and measures of statistical heterogeneity. If comparing groups, describe the direction of the effect.	N/A
	20c	Present results of all investigations of possible causes of heterogeneity among study results.	N/A

Section and Topic	Item #	Checklist item	Location where item is reported
	20d	Present results of all sensitivity analyses conducted to assess the robustness of the synthesized results.	N/A
Reporting biases	21	Present assessments of risk of bias due to missing results (arising from reporting biases) for each synthesis assessed.	N/A
Certainty of evidence	22	Present assessments of certainty (or confidence) in the body of evidence for each outcome assessed.	N/A
**DISCUSSION**			
Discussion	23a	Provide a general interpretation of the results in the context of other evidence.	Table 3
	23b	Discuss any limitations of the evidence included in the review.	Pages 17-18, 25-27
	23c	Discuss any limitations of the review processes used.	Page 33-34
	23d	Discuss implications of the results for practice, policy, and future research.	Page 31
**OTHER INFORMATION**			
Registration and protocol	24a	Provide registration information for the review, including register name and registration number, or state that the review was not registered.	Page 8
	24b	Indicate where the review protocol can be accessed, or state that a protocol was not prepared.	Page 8
	24c	Describe and explain any amendments to information provided at registration or in the protocol.	N/A
Support	25	Describe sources of financial or non-financial support for the review, and the role of the funders or sponsors in the review.	N/A
Competing interests	26	Declare any competing interests of review authors.	N/A
Availability of data, code and other materials	27	Report which of the following are publicly available and where they can be found: template data collection forms; data extracted from included studies; data used for all analyses; analytic code; any other materials used in the review.	N/A

Table S2. PRISMA 2020 for abstracts checklist

Section and Topic	Item #	Checklist item	Reported (Yes/No)
**TITLE**			
Title	1	Identify the report as a systematic review.	Yes
**BACKGROUND**			
Objectives	2	Provide an explicit statement of the main objective(s) or question(s) the review addresses.	Yes
**METHODS**			
Eligibility criteria	3	Specify the inclusion and exclusion criteria for the review.	Yes
Information sources	4	Specify the information sources (e.g. databases, registers) used to identify studies and the date when each was last searched.	Yes
Risk of bias	5	Specify the methods used to assess risk of bias in the included studies.	Yes
Synthesis of results	6	Specify the methods used to present and synthesise results.	Yes
**RESULTS**			
Included studies	7	Give the total number of included studies and participants and summarise relevant characteristics of studies.	Yes
Synthesis of results	8	Present results for main outcomes, preferably indicating the number of included studies and participants for each. If meta-analysis was done, report the summary estimate and confidence/credible interval. If comparing groups, indicate the direction of the effect (i.e. which group is favoured).	Yes
**DISCUSSION**			
Limitations of evidence	9	Provide a brief summary of the limitations of the evidence included in the review (e.g. study risk of bias, inconsistency and imprecision).	Yes
Interpretation	10	Provide a general interpretation of the results and important implications.	Yes
**OTHER**			
Funding	11	Specify the primary source of funding for the review.	N/A
Registration	12	Provide the register name and registration number.	Yes

Table S3. Medline search strategy

Line #	Search term(s)
1	vaccination refusal/ or vaccination hesitancy/
2	Anti-Vaccination Movement/
3	(antivaccin* or anti-vaccin* or anti-immuniz*).ti,ab,kf.
4	((immuniz* or vaccin*) adj3 (hesit* or refus* or delay* or uptak* or accept*)).ti,ab,kf.
5	or/1-4
6	coronavirus infections/ or covid-19/
7	coronavirus/ or betacoronavirus/ or sars-cov-2/
8	exp COVID-19 Vaccines/
9	(covid or covid-19 or covid19 or SARS-COV-2 or coronavirus* or corona virus*).ti,ab,kf.
10	or/6-9
11	5 and 10
12	limit 12 to yr="2019-current"

Table S4. Embase search strategy

Line #	Search term(s)
1	vaccination refusal/ or vaccination hesitancy/
2	Anti-Vaccination Movement/
3	(antivaccin* or anti-vaccin* or anti-immuniz*).ti,ab,kw.
4	((immuniz* or vaccin*) adj3 (hesit* or refus* or delay* or uptak* or accept*)).ti,ab,kw.
5	or/1-4
6	coronavirus infections/ or covid-19/
7	coronavirus/ or betacoronavirus/ or sars-cov-2/
8	exp COVID-19 Vaccines/
9	(covid or covid-19 or covid19 or SARS-COV-2 or coronavirus* or corona virus*).ti,ab,kw.
10	or/6-9
11	5 and 10
12	limit 12 to yr="2019-current"

Table S5. PsycInfo search strategy

Line #	Search term(s)
1	(antivaccin* or anti-vaccin* or anti-immuniz*).mp. [mp=title, abstract, heading word, table of contents, key concepts, original title, tests & measures, mesh word]
2	((immuniz* or vaccin*) adj3 (hesit* or refus* or delay* or uptak* or accept*)).mp. [mp=title, abstract, heading word, table of contents, key concepts, original title, tests & measures, mesh word]
3	1 or 2
4	covid-19/ or coronavirus/ or epidemics/ or pandemics/ or public health/
5	(covid or covid-19 or covid19 or SARS-COV-2 or coronavirus* or corona virus*).mp. [mp=title, abstract, heading word, table of contents, key concepts, original title, tests & measures, mesh word]
6	4 or 5
7	3 and 6
8	limit 7 to yr="2019 -Current"

Table S6. CINAHL search strategy

Line #	Search term(s)
1	(MH "Anti-Vaccination Movement")
2	TI ((antivaccin* or anti-vaccin* or anti-immuniz*)) OR AB ((antivaccin* or anti-vaccin* or anti-immuniz*))
3	TI (((immuniz* or vaccin*) N3 (hesit* or refus* or delay* or uptak* or accept*))) OR AB (((immuniz* or vaccin*) N3 (hesit* or refus* or delay* or uptak* or accept*)))
4	S1 OR S2 OR S3
5	(MH "COVID-19") OR (MH "Coronavirus Infections")
6	(MH "COVID-19 Vaccines")
7	(MH "COVID-19 Pandemic")
8	(MH "SARS-CoV-2") OR (MH "Coronavirus")
9	TI ((covid or covid-19 or covid19 or SARS-COV-2 or coronavirus* or corona virus*)) OR AB ((covid or covid-19 or covid19 or SARS-COV-2 or coronavirus* or corona virus*))
10	S5 OR S6 OR S7 OR S8 OR S9
11	S4 and S10
12	Published Date: 20200101-20221231
13	Peer reviewed

Table S7. Web of Science search strategy

Line #	Search term(s)
1	(antivaccin* or anti-vaccin* or anti-immuniz*) (Title) or (antivaccin* or anti-vaccin* or anti-immuniz*) (Abstract) or (antivaccin* or anti-vaccin* or anti-immuniz*) (Author Keywords)
2	((immuniz* or vaccin*) NEAR/3 (hesit* or refus* or delay* or uptak* or accept*)) (Title)or ((immuniz* or vaccin*) NEAR/3 (hesit* or refus* or delay* or uptak* or accept*))(Abstract) or ((immuniz* or vaccin*) NEAR/3 (hesit* or refus* or delay* or uptak* or accept*)) (Author Keywords)
3	#1 or #2
4	(covid or covid-19 or covid19 or SARS-COV-2 or coronavirus* or corona virus*) (Title)or (covid or covid-19 or covid19 or SARS-COV-2 or coronavirus* or corona virus*)(Abstract) or (covid or covid-19 or covid19 or SARS-COV-2 or coronavirus* or corona virus*) (Author Keywords)
5	#3 and #4
6	#5 publication date 2020-01-01 to 2022-12-31

Table S8. Articles excluded during full-text review and reasons for exclusion

Reference	Reason for exclusion
Abdel-Qader, D. H., Hayajneh, W., Albassam, A., Obeidat, N. M., Belbeisi, A. M., Al Mazrouei, N., Al-Shaikh, A. F., Nusair, K. E., Al Meslamani, A. Z., El-Shara, A. A., El Sharu, H., Mohammed Ebaed, S. B., & Mohamed Ibrahim, O. (2022). Pharmacists-physicians collaborative intervention to reduce vaccine hesitancy and resistance: A randomized controlled trial. *Vaccine, 10*, 100135. https://doi.org/10.1016/j.jvacx.2021.100135	Ineligible outcome (e.g., measures of vaccine hesitancy, vaccine intentions)
Abdel-Qader, D. H., Al Meslamani, A. Z., Al Mazrouei, N., El-Shara, A. A., El Sharu, H., Merghani Ali, E., Mohammed Ebaed, S. B., & Mohamed Ibrahim, O. (2021). Virtual Coaching Delivered by Pharmacists to Prevent COVID-19 Transmission. *Hospital Pharmacy*, *57*(2), 300–308. https://doi.org/10.1177/00185787211032354	Ineligible research question (does not evaluate the effectiveness of a non-financial intervention for COVID-19 vaccine hesitancy)
Abdul-Mutakabbir, J., Casey, S., Jews, V., King, A., Simmons, K., Peteet, B., Belliard, J. C., Hogue, M., & Peverini, R. (2021). The Utility of Community-Academic Partnerships in Promoting the Equitable Delivery of COVID-19 Vaccines in Black Communities. *Open Forum Infectious Diseases*, *8*(1), S339–S339. https://doi.org/10.1093/ofid/ofab466.674	Ineligible publication type (e.g., editorial/commentary, abstract with insufficient information, protocol, review article)
Aborode, A. T., Fajemisin, E. A., Ekwebelem, O. C., Tsagkaris, C., Taiwo, E. A., Uwishema, O., Awoniyi, O. O., Ahmad, S., Essar, M. Y., Adanur, I., & Yunusa, I. (2021). Vaccine hesitancy in Africa: causes and strategies to the rescue. *Therapeutic Advances in Vaccines and Immunotherapy*, *9*, 251513552110475. https://doi.org/10.1177/25151355211047514	Ineligible publication type (e.g., editorial/commentary, abstract with insufficient information, protocol, review article)
Abou Leila, R., Salamah, M., & El-Nigoumi, S. (2021). Reducing COVID-19 Vaccine Hesitancy by Implementing Organizational Intervention in a Primary Care Setting in Bahrain. *Cureus*. https://doi.org/10.7759/cureus.19282	Ineligible outcome (e.g., feelings of vaccine hesitancy, measures of vaccine intentions)
Abrams, E. M., Shaker, M., Sinha, I., & Greenhawt, M. (2021). COVID-19 vaccines: addressing hesitancy in young people with allergies. *The Lancet Respiratory Medicine*, *9*(10), 1090–1092. https://doi.org/10.1016/s2213-2600(21)00370-2	Ineligible publication type (e.g., editorial/commentary, abstract with insufficient

	information, protocol, review article)
Acar-Burkay, S., & Cristian, D. C. (2022). Cognitive underpinnings of COVID-19 vaccine hesitancy. *Social Science & Medicine*, *301*, 114911. https://doi.org/10.1016/j.socscimed.2022.114911	Ineligible research question (does not evaluate the effectiveness of a non-financial intervention for COVID-19 vaccine hesitancy)
Acharya, B., & Dhakal, C. (2021). Implementation of State Vaccine Incentive Lottery Programs and Uptake of COVID-19 Vaccinations in the United States. *JAMA Network Open*, *4*(12), e2138238. https://doi.org/10.1001/jamanetworkopen.2021.38238	Ineligible research question (does not evaluate the effectiveness of a non-financial intervention for COVID-19 vaccine hesitancy)
Ahmed, N., Boxley, C., Dixit, R., Krevat, S., Fong, A., Ratwani, R. M., & Wesley, D. B. (2022). Evaluation of a Text Message–Based COVID-19 Vaccine Outreach Program Among Older Patients: Cross-sectional Study. *JMIR Formative Research*, *6*(7), e33260. https://doi.org/10.2196/33260	Ineligible outcome (e.g., feelings of vaccine hesitancy, measures of vaccine intentions)
Aida, T., & Shoji, M. (2022). Cross-country evidence on the role of national governance in boosting COVID-19 vaccination. *BMC Public Health*, *22*(1). https://doi.org/10.1186/s12889-022-12985-5	Ineligible research question (does not evaluate the effectiveness of a non-financial intervention for COVID-19 vaccine hesitancy)
Ajeigbe, O., Arage, G., Besong, M., Chacha, W., Desai, R., Doegah, P., Hamoonga, T. E., Hussein, H., Matchado, A., Mbotwe-Sibanda, S., Mukoma, G., Odebode, A., Olawole, T., Phaswana, M., Rotimi, O., Silubonde, T. M., Thabethe, N., Thiba, A., Thomford, N. E., . . . Macnab, A. (2022). Culturally relevant COVID-19 vaccine acceptance strategies in sub-Saharan Africa. *The Lancet Global Health*, *10*(8), e1090–e1091. https://doi.org/10.1016/s2214-109x(22)00251-0	Ineligible publication type (e.g., editorial/commentary, abstract with insufficient information, protocol, review article)
Akpoji, U., Amos, M. E., McMillan, K., Sims, S., & Rife, K. (2022). Exercising empathy: Pharmacists possess skills to increase coronavirus vaccine confidence. *Journal of the American Pharmacists Association*, *62*(1), 296–301. https://doi.org/10.1016/j.japh.2021.07.016	Ineligible publication type (e.g., editorial/commentary, abstract with insufficient

	information, protocol, review article)
Ala, A., Edge, C., Zumla, A., & Shafi, S. (2021). Specific COVID-19 messaging targeting ethnic minority communities. *EClinicalMedicine*, *35*, 100862. https://doi.org/10.1016/j.eclinm.2021.100862	Ineligible publication type (e.g., editorial/commentary, abstract with insufficient information, protocol, review article)
Ali, N., Ashiru-Oredope, D., & Murdan, S. (2021). Training university students as vaccination champions to promote vaccination in their multiple identities and help address vaccine hesitancy. *Pharmacy Education*, *21*, 407–419. https://doi.org/10.46542/pe.2021.211.407419	Ineligible outcome (e.g., feelings of vaccine hesitancy, measures of vaccine intentions)
Altay, S., Hacquin, A. S., Chevallier, C., & Mercier, H. (2021). Information delivered by a chatbot has a positive impact on COVID-19 vaccines attitudes and intentions. *Journal of Experimental Psychology: Applied*. https://doi.org/10.1037/xap0000400	Ineligible outcome (e.g., feelings of vaccine hesitancy, measures of vaccine intentions)
Ambale, C., & Murerwa, C. (2021). Leveraging on community health volunteers to educate the public on adverse events following immunization and dispel myths and misconceptions. *Drug Safety*, *44*(12). https://doi.org/10.1007/s40264-021-01129-0	Ineligible publication type (e.g., editorial/commentary, abstract with insufficient information, protocol, review article)
Barber, A., & West, J. (2022). Conditional cash lotteries increase COVID-19 vaccination rates. *Journal of Health Economics*, *81*, 102578. https://doi.org/10.1016/j.jhealeco.2021.102578	Ineligible research question (does not evaluate the effectiveness of a non-financial intervention for COVID-19 vaccine hesitancy)
Bares, S. H., Carr, R., Jones, S. L., Regan, N. N., Orduna, V., Cramer, D. W., Tran, C. D., & Fadul, N. (2021). A Multi-faceted, Iterative Program to Increase COVID-19 Vaccine Uptake in a Midwestern HIV Clinic. *Open Forum Infectious Diseases*, *8*(Supplement_1), S688–S689. https://doi.org/10.1093/ofid/ofab466.1388	Ineligible publication type (e.g., editorial/commentary, abstract with insufficient information, protocol, review article)

Barnes, K., & Colagiuri, B. (2022). Positive Attribute Framing Increases COVID-19 Booster Vaccine Intention for Unfamiliar Vaccines. *Vaccines*, *10*(6), 962. https://doi.org/10.3390/vaccines10060962	Ineligible outcome (e.g., feelings of vaccine hesitancy, measures of vaccine intentions)
Berliner Senderey, A., Ohana, R., Perchik, S., Erev, I., & Bailer, R. D. (2022). Encouraging COVID-19 vaccination through behaviorally informed reminders. *Behavioural Science & Policy*, *8*(1). https://doi.org/10.2139/ssrn.3852345	Ineligible outcome (e.g., feelings of vaccine hesitancy, measures of vaccine intentions)
Berrou, I., Hamilton, K., Cook, C., Armour, C., Hughes, S., Hancock, J., Quigg, S., Hajinur, H., Srivastava, S., Kenward, C., Ali, A., Hobbs, L., Milani, E., & Walsh, N. (2022). Leaving No One Behind: Interventions and Outcomes of the COVID-19 Vaccine Maximising Uptake Programme. *Vaccines*, *10*(6), 840. https://doi.org/10.3390/vaccines10060840	No eligible comparator (i.e., control group)
Bischof, J., Schoeffler, A., Bashian, E., Callender, N., Fuentes, A., Geyer, E., More, A., Webb, T., & Kman, K. (2021). Implementation of a COVID-19 Vaccine Emergency Department Education Program for Underserved Communities: A Pilot Quality Improvement Project. *Annals of Emergency Medicine*, *78*(2), S13. https://doi.org/10.1016/j.annemergmed.2021.07.027	Ineligible outcome (e.g., feelings of vaccine hesitancy, measures of vaccine intentions)
Boguslavsky, D. V., Sharov, K. S., & Sharova, N. P. (2022). Counteracting conspiracy ideas as a measure of increasing propensity for COVID-19 vaccine uptake in Russian society. *Journal of Global Health*, *2*. https://doi.org/10.7189/jogh.12.03013	Ineligible publication type (e.g., editorial/commentary, abstract with insufficient information, protocol, review article)
Bokemper, S. E., Gerber, A. S., Omer, S. B., & Huber, G. A. (2021). Persuading US White evangelicals to vaccinate for COVID-19: Testing message effectiveness in fall 2020 and spring 2021. *Proceedings of the National Academy of Sciences*, *118*(49), e2114762118. https://doi.org/10.1073/pnas.2114762118	Ineligible outcome (e.g., feelings of vaccine hesitancy, measures of vaccine intentions)
Budhwani, H., Sharma, V., Long, D., & Simpson, T. (2022). Developing a Clinic-Based, Vaccine-Promoting Intervention for African American Youth in Rural Alabama: Protocol for a Pilot Cluster-Randomized Controlled Implementation Science Trial. *JMIR Research Protocols*, *11*(4), e33982. https://doi.org/10.2196/33982	Ineligible publication type (e.g., editorial/commentary, abstract with insufficient information, protocol, review article)

Burkhardt, M. C., Real, F. J., DeBlasio, D., Beck, A. F., Reyner, A., & Rosen, B. L. (2022). Increasing Coronavirus Disease 2019 Vaccine Uptake in Pediatric Primary Care by Offering Vaccine to Household Members. *The Journal of Pediatrics*, *247*, 150-154.e1. https://doi.org/10.1016/j.jpeds.2022.04.023	No eligible comparator (i.e., control group)
Campos-Mercade, P., Meier, A. N., Schneider, F. H., Meier, S., Pope, D., & Wengström, E. (2021). Monetary incentives increase COVID-19 vaccinations. *Science*, *374*(6569), 879–882. https://doi.org/10.1126/science.abm0475	Ineligible research question (does not evaluate the effectiveness of a non-financial intervention for COVID-19 vaccine hesitancy)
Carlson, S. J., McKenzie, L., Roberts, L., Blyth, C. C., & Attwell, K. (2022). Does a major change to a COVID-19 vaccine program alter vaccine intention? A qualitative investigation. *Vaccine*, *40*(4), 594–600. https://doi.org/10.1016/j.vaccine.2021.12.021	Ineligible research question (does not evaluate the effectiveness of a non-financial intervention for COVID-19 vaccine hesitancy)
Cheng, T., Kreitman, K., Stowe, C., Waters, B., & Baidoo, L. (2022). Improving the vaccination rates for immunosuppressed patients with inflammatory bowel disease. *Inflammatory Bowel Diseases*, *28*(Supplement_1), S84–S85. https://doi.org/10.1093/ibd/izac015.136	Ineligible publication type (e.g., editorial/commentary, abstract with insufficient information, protocol, review article)
Chevallier, C., Hacquin, A. S., & Mercier, H. (2021). COVID-19 Vaccine Hesitancy: Shortening the Last Mile. *Trends in Cognitive Sciences*, *25*(5), 331–333. https://doi.org/10.1016/j.tics.2021.02.002	Ineligible publication type (e.g., editorial/commentary, abstract with insufficient information, protocol, review article)
Cookson, D., Jolley, D., Dempsey, R. C., & Povey, R. (2021). A social norms approach intervention to address misperceptions of anti-vaccine conspiracy beliefs amongst UK parents. *PLOS ONE*, *16*(11), e0258985. https://doi.org/10.1371/journal.pone.0258985	Ineligible outcome (e.g., feelings of vaccine hesitancy, measures of vaccine intentions)
Cordero, D. A. (2022). Exploring interventions against COVID-19 vaccine hesitancy in the Philippines. *Clinical and Experimental Vaccine Research*, *11*(2), 233. https://doi.org/10.7774/cevr.2022.11.2.233	Ineligible publication type (e.g., editorial/commentary,

	abstract with insufficient information, protocol, review article)
de Vere Hunt, I., Dunn, T., Mahoney, M., Chen, M., Zhang, L., Bousheri, S., Bernard, D., & Linos, E. (2021). A social media-based campaign to promote COVID-19 vaccine uptake in underrepresented groups in the US. *European Journal of Public Health*, *31*. https://doi.org/10.1093/eurpub/ckab165.170	Ineligible publication type (e.g., editorial/commentary, abstract with insufficient information, protocol, review article)
Dhif, Y., Bonnabry, P., & Diana, A. (2022). To vaccinate or not to vaccinate: impact of a public health action on vaccine hesitancy. *European Journal of Hospital Pharmacy*, *29*, A181. https://doi.org/10.1136/ejhpharm-2022-eahp.380	Ineligible publication type (e.g., editorial/commentary, abstract with insufficient information, protocol, review article)
Di Mauro, A., Di Mauro, F., De Nitto, S., Rizzo, L., Greco, C., Stefanizzi, P., Tafuri, S., Baldassarre, M. E., & Laforgia, N. (2022). Social Media Interventions Strengthened COVID-19 Immunization Campaign. *Frontiers in Pediatrics*, *10*. https://doi.org/10.3389/fped.2022.869893	Ineligible population (e.g., pediatric population only)
Diaz, D., Chacko, S., Sperling, A., Fleck, E., Louh, I., Trepp, R., & Ye, S. (2022). Assessment of Digital and Community-Based Outreach Interventions to Encourage COVID-19 Vaccination Uptake in an Underserved Community. *JAMA Network Open*, *5*(6), e2217875. https://doi.org/10.1001/jamanetworkopen.2022.17875	Ineligible outcome (e.g., feelings of vaccine hesitancy, measures of vaccine intentions)
Eissa, A., Lofters, A., Akor, N., Prescod, C., & Nnorom, O. (2021). Increasing SARS-CoV-2 vaccination rates among Black people in Canada. *Canadian Medical Association Journal*, *193*(31), E1220–E1221. https://doi.org/10.1503/cmaj.210949	Ineligible publication type (e.g., editorial/commentary, abstract with insufficient information, protocol, review article)
L. Ferrer-Gonzalez, G. Falconi-Adame, Shivani Priyadarshni, D. Benyo, D. High, K. Pejo, A. Yechuri, Ruben Perez, & Bryan Curry. (2019). Combating inpatient vaccine hesitancy: I raised the rates in my cardiac patients. *Circulation-Cardiovascular Quality and Outcomes*, *13*. https://scholarlycommons.hcahealthcare.com/cardiology/44/	Ineligible publication type (e.g., editorial/commentary, abstract with insufficient information, protocol, review article)

Ford, K. L., West, A. B., Bucher, A., & Osborn, C. Y. (2022). Personalized Digital Health Communications to Increase COVID-19 Vaccination in Underserved Populations: A Double Diamond Approach to Behavioral Design. *Frontiers in Digital Health*, *4*. https://doi.org/10.3389/fdgth.2022.831093	Ineligible research question (does not evaluate the effectiveness of a non-financial intervention for COVID-19 vaccine hesitancy)
Freeman, D., Loe, B. S., Yu, L. M., Freeman, J., Chadwick, A., Vaccari, C., Shanyinde, M., Harris, V., Waite, F., Rosebrock, L., Petit, A., Vanderslott, S., Lewandowsky, S., Larkin, M., Innocenti, S., Pollard, A. J., McShane, H., & Lambe, S. (2021). Effects of different types of written vaccination information on COVID-19 vaccine hesitancy in the UK (OCEANS-III): a single-blind, parallel-group, randomised controlled trial. *The Lancet Public Health*, *6*(6), e416–e427. https://doi.org/10.1016/s2468-2667(21)00096-7	Ineligible outcome (e.g., feelings of vaccine hesitancy, measures of vaccine intentions)
Fujita, A., Goolsby, T., Powell, K., & Cartwright, E. J. (2021). 587. An Intervention to Improve COVID-19 Vaccination Rates Among Inpatients at a Veterans Affairs Hospital. *Open Forum Infectious Diseases*, *8*(Supplement_1), S396–S396. https://doi.org/10.1093/ofid/ofab466.785	Ineligible publication type (e.g., editorial/commentary, abstract with insufficient information, protocol, review article)
Gadarian, S. K., Goodman, S. W., Michener, J., Nyhan, B., & Pepinsky, T. B. (2022). Information From Same-Race/Ethnicity Experts Online Does Not Increase Vaccine Interest or Intention to Vaccinate. *The Milbank Quarterly*, *100*(2), 492–503. https://doi.org/10.1111/1468-0009.12561	Ineligible outcome (e.g., feelings of vaccine hesitancy, measures of vaccine intentions)
Ganczak, M., Pasek, O., Duda-Duma, U., Komorzycka, J., Nowak, K., & Korzeń, M. (2021). A Peer-Based Educational Intervention Effects on SARS-CoV-2 Knowledge and Attitudes among Polish High-School Students. *International Journal of Environmental Research and Public Health*, *18*(22), 12183. https://doi.org/10.3390/ijerph182212183	Ineligible population (e.g., pediatric population only)
Gebrezghi, S., Muffly, T., Schultz, C., & Larrea, N. (2022). Preoperative counseling regarding COVID-19 vaccination. *American Journal of Obstetrics and Gynecology*, *226*(3), S1364–S1365. https://doi.org/10.1016/j.ajog.2021.12.213	Ineligible publication type (e.g., editorial/commentary, abstract with insufficient information, protocol, review article)
Georgiou, A., Chang, J., & Karaca-Mandic, P. (2022). Association of Large Financial Incentives With COVID-19 Vaccination Uptake Among	No eligible comparator (i.e., control group)

Employees of a Large Private Company. *JAMA Network Open*, *5*(4), e229812. https://doi.org/10.1001/jamanetworkopen.2022.9812	
Glendening, J., Bickford, B., Markert, R., Yuhas, J., Berglund, A., Kelly, D., Scott, J., & Burtson, K. (2022). Addressing Persistent Vaccine Hesitancy in a Military Community Through a Physician-Led Intervention. *Military Medicine*. https://doi.org/10.1093/milmed/usac176	Ineligible outcome (e.g., feelings of vaccine hesitancy, measures of vaccine intentions)
Gonzalez, C. J., Meltzer, K., Jabri, A., Zhu, J. J., Lau, J. D., Pelzman, F., & Tung, J. (2022). Development of a Practice-based Community Outreach Intervention to Prevent Inequities in COVID-19 Vaccinations. *American Journal of Medical Quality*, *37*(4), 348–355. https://doi.org/10.1097/jmq.0000000000000049	Ineligible outcome (e.g., feelings of vaccine hesitancy, measures of vaccine intentions)
Goren, A., Lanyado, A., Yesharim, R., Wolk, D. M., Doyle, J., Meyer, M. N., & Chabris, C. F. (2021). Informing patients that they are at high risk for serious complications of viral infection increases vaccination rates. *MedRxiv*. https://doi.org/10.1101/2021.02.20.21252015	Ineligible research question (does not evaluate the effectiveness of a non-financial intervention for COVID-19 vaccine hesitancy)
Hirshberg, J. S., Huysman, B. C., Oakes, M. C., Cater, E. B., Odibo, A. O., Raghuraman, N., & Kelly, J. C. (2021). Offering onsite COVID-19 vaccination to high-risk obstetrical patients: initial findings. *American Journal of Obstetrics & Gynecology MFM*, *3*(6), 100478. https://doi.org/10.1016/j.ajogmf.2021.100478	Ineligible research question (does not evaluate the effectiveness of a non-financial intervention for COVID-19 vaccine hesitancy)
Huang, Y., & Green, M. C. (2022). Reducing COVID-19 vaccine hesitancy among African Americans: the effects of narratives, character's self-persuasion, and trust in science. *Journal of Behavioral Medicine*. https://doi.org/10.1007/s10865-022-00303-8	Ineligible outcome (e.g., feelings of vaccine hesitancy, measures of vaccine intentions)
Hulen, E., Rynerson, A. L., & Dobscha, S. K. (2022). Vaccine hesitancy among Veterans Affairs Health Care System employees. *Preventive Medicine Reports*, *26*, 101702. https://doi.org/10.1016/j.pmedr.2022.101702	No full text available
Iyer, G., Nandur, V., & Soberman, D. (2022). Vaccine hesitancy and monetary incentives. *Humanities and Social Sciences Communications*, *9*(1). https://doi.org/10.1057/s41599-022-01074-y	Ineligible research question (does not evaluate the effectiveness of a non-financial

	intervention for COVID-19 vaccine hesitancy)
Jensen, U. T., Ayers, S., & Koskan, A. M. (2022). Video-based messages to reduce COVID-19 vaccine hesitancy and nudge vaccination intentions. *PLOS ONE*, *17*(4), e0265736. https://doi.org/10.1371/journal.pone.0265736	Ineligible outcome (e.g., feelings of vaccine hesitancy, measures of vaccine intentions)
Joska, J. A., Rabie, S., & Sibeko, G. (n.d.). Changing minds: A behavioural approach to vaccine hesitancy. *South African Medical Journal*, *112*(2), 66–67. https://doi.org/10.7196/SAMJ.2022.v112i2.16301	Ineligible publication type (e.g., editorial/commentary, abstract with insufficient information, protocol, review article)
Kachurka, R., Krawczyk, M., & Rachubik, J. (2021). Persuasive Messages Will Not Increase COVID-19 Vaccine Acceptance: Evidence from a Nationwide Online Experiment. *Vaccines*, *9*(10), 1113. https://doi.org/10.3390/vaccines9101113	Ineligible outcome (e.g., feelings of vaccine hesitancy, measures of vaccine intentions)
Kassi, L. A., Swanson, A., Lawson, A. K., Shah, S., & Pavone, M. E. (2022). Psychological distress in women considering or undergoing fertility treatments during the omicron surge of the COVID-19 pandemic. *Fertility and Sterility*, *118*(4), e313. https://doi.org/10.1016/j.fertnstert.2022.09.077	Ineligible publication type (e.g., editorial/commentary, abstract with insufficient information, protocol, review article)
Kelkar, A. H., Blake, J. A., Cherabuddi, K., Cornett, H., McKee, B. L., & Cogle, C. R. (2021). Vaccine Enthusiasm and Hesitancy in Cancer Patients and the Impact of a Webinar. *Healthcare*, *9*(3), 351. https://doi.org/10.3390/healthcare9030351	Ineligible outcome (e.g., feelings of vaccine hesitancy, measures of vaccine intentions)
Kharbanda, E. O., & Vazquez-Benitez, G. (2022). COVID-19 mRNA Vaccines During Pregnancy. *JAMA*, *327*(15), 1451. https://doi.org/10.1001/jama.2022.2459	Ineligible publication type (e.g., editorial/commentary, abstract with insufficient information, protocol, review article)
Kim, M. H., Son, N. H., Park, Y. S., Lee, J. H., Kim, D. A., & Kim, Y. C. (2021). Effect of a hospital-wide campaign on COVID-19 vaccination uptake among healthcare workers in the context of raised concerns for life-	No eligible comparator (i.e., control group)

threatening side effects. *PLOS ONE*, *16*(10), e0258236. https://doi.org/10.1371/journal.pone.0258236	
Knight, H., Jia, R., Ayling, K., Bradbury, K., Baker, K., Chalder, T., Morling, J., Durrant, L., Avery, T., Ball, J., Barker, C., Bennett, R., McKeever, T., & Vedhara, K. (2021). Understanding and addressing vaccine hesitancy in the context of COVID-19: development of a digital intervention. *Public Health*, *201*, 98–107. https://doi.org/10.1016/j.puhe.2021.10.006	Ineligible research question (does not evaluate the effectiveness of a non-financial intervention for COVID-19 vaccine hesitancy)
Knudson, J., Gharib, S., & Phillips, A. (n.d.). Overcoming COVID vaccine hesitancy in our college athlete population. *Clinical Journal of Sport Medicine*, *32*(2). https://doi.org/10.1097/JSM.0000000000001014	Ineligible publication type (e.g., editorial/commentary, abstract with insufficient information, protocol, review article)
Lang, D., Esbenshade, L., & Willer, R. (2022). Did Ohio's Vaccine Lottery Increase Vaccination Rates? A Pre-Registered, Synthetic Control Study. *Journal of Experimental Political Science*, 1–19. https://doi.org/10.1017/xps.2021.32	Ineligible research question (does not evaluate the effectiveness of a non-financial intervention for COVID-19 vaccine hesitancy)
Lazić, A., Kalinova, K. N., Packer, J., Pae, R., Petrović, M. B., Popović, D., Sievert, D. E. C., & Stafford-Johnson, N. (2021). Social nudges for vaccination: How communicating herd behaviour influences vaccination intentions. *British Journal of Health Psychology*, *26*(4), 1219–1237. https://doi.org/10.1111/bjhp.12556	Ineligible outcome (e.g., feelings of vaccine hesitancy, measures of vaccine intentions)
Lee, D., Rundle-Thiele, S., & Li, G. (2021). Motivating Seasonal Influenza Vaccination and Cross-Promoting COVID-19 Vaccination: An Audience Segmentation Study among University Students. *Vaccines*, *9*(12), 1397. https://doi.org/10.3390/vaccines9121397	Ineligible research question (does not evaluate the effectiveness of a non-financial intervention for COVID-19 vaccine hesitancy)
Li, P. C., Theis, S. R., Kelly, D., Ocampo, T., Berglund, A., Morgan, D., Markert, R., Fisher, E., & Burtson, K. (2021). Impact of an Education Intervention on COVID-19 Vaccine Hesitancy in a Military Base Population. *Military Medicine*, *187*(11–12), e1449–e1455. https://doi.org/10.1093/milmed/usab363	Ineligible outcome (e.g., feelings of vaccine hesitancy, measures of vaccine intentions)

Li, S., Xia, Y., Zhao, W., Miao, X., & Xu, Q. (2022). Self-affirmation increases acceptance of information on COVID-19 vaccines and promotes vaccination intention. *Journal of Behavioral Medicine*. https://doi.org/10.1007/s10865-022-00292-8	Ineligible outcome (e.g., feelings of vaccine hesitancy, measures of vaccine intentions)
Lim, C., Van Alphen, M. U., Maclaurin, S., Mulligan, C., Macri, B., Cather, C., & Freudenreich, O. (2022). Increasing COVID-19 Vaccination Rates Among Patients With Serious Mental Illness: A Pilot Intervention Study. *Psychiatric Services*, *73*(11), 1274–1277. https://doi.org/10.1176/appi.ps.202100702	No eligible comparator (i.e., control group)
Mahase, E. (2021). Covid-19 vaccines: GPs boost uptake by calling patients and teaming up with community groups. *BMJ*, n2093. https://doi.org/10.1136/bmj.n2093	Ineligible publication type (e.g., editorial/commentary, abstract with insufficient information, protocol, review article)
Mallow, P. J., Enis, A., Wackler, M., & Hooker, E. A. (2022). COVID-19 financial lottery effect on vaccine hesitant areas: Results from Ohio's Vax-a-million program. *The American Journal of Emergency Medicine*, *56*, 316–317. https://doi.org/10.1016/j.ajem.2021.08.053	Ineligible research question (does not evaluate the effectiveness of a non-financial intervention for COVID-19 vaccine hesitancy)
Mandelbaum, J. (2022). Worth Slowing Down for: Road Signs and Messages About the COVID-19 Pandemic. *American Journal of Public Health*, *112*(3), 391–392. https://doi.org/10.2105/ajph.2021.306626	Ineligible publication type (e.g., editorial/commentary, abstract with insufficient information, protocol, review article)
Marquez, C., Kerkhoff, A. D., Naso, J., Contreras, M. G., Castellanos Diaz, E., Rojas, S., Peng, J., Rubio, L., Jones, D., Jacobo, J., Rojas, S., Gonzalez, R., Fuchs, J. D., Black, D., Ribeiro, S., Nossokoff, J., Tulier-Laiwa, V., Martinez, J., Chamie, G., . . . Havlir, D. V. (2021). A multi-component, community-based strategy to facilitate COVID-19 vaccine uptake among Latinx populations: From theory to practice. *PLOS ONE*, *16*(9), e0257111. https://doi.org/10.1371/journal.pone.0257111	Ineligible publication type (e.g., editorial/commentary, abstract with insufficient information, protocol, review article)
Mažar, N. (2022). Give physicians' views to improve COVID vaccine uptake. *Nature*, *606*(7914), 471–472. https://doi.org/10.1038/d41586-022-01305-x	Ineligible publication type (e.g., editorial/commentary, abstract with

	insufficient information, protocol, review article)
Mena Lora, A. J., Echeverria, S. L., Li, E., Morales, M., Esquiliano, R., Schultz, G., Sifuentes, J., Spencer, S., Takhsh, E., & Lavani, R. (2021). 566. Impact of a Culturally Sensitive Multilingual Community Outreach Model on COVID-19 Vaccinations at an Urban Safety-net Community Hospital. *Open Forum Infectious Diseases*, *8*(Supplement_1), S385–S385. https://doi.org/10.1093/ofid/ofab466.764	Ineligible publication type (e.g., editorial/commentary, abstract with insufficient information, protocol, review article)
Moberly, T. (2021). Covid-19: Vaccine hesitancy fell after vaccination programme started. *BMJ*, n837. https://doi.org/10.1136/bmj.n837	Ineligible publication type (e.g., editorial/commentary, abstract with insufficient information, protocol, review article)
Mottelson, A., Vandeweerdt, C., Atchapero, M., Luong, T., Holz, C., Böhm, R., & Makransky, G. (2021). A self-administered virtual reality intervention increases COVID-19 vaccination intention. *Vaccine*, *39*(46), 6746–6753. https://doi.org/10.1016/j.vaccine.2021.10.004	Ineligible outcome (e.g., feelings of vaccine hesitancy, measures of vaccine intentions)
Okuhara, T., Okada, H., Goto, E., Tsunezumi, A., Kagawa, Y., & Kiuchi, T. (2022). Encouraging COVID-19 vaccination via an evolutionary theoretical approach: A randomized controlled study in Japan. *Patient Education and Counseling*, *105*(7), 2248–2255. https://doi.org/10.1016/j.pec.2022.02.013	Ineligible outcome (e.g., feelings of vaccine hesitancy, measures of vaccine intentions)
Onigbinde, O. A., & Ajagbe, A. O. (2022). COVID-19 vaccination and herd immunity In Africa: An incentive-based approach could be the game-changer to vaccine hesitancy. *Public Health in Practice*, *4*, 100282. https://doi.org/10.1016/j.puhip.2022.100282	Ineligible publication type (e.g., editorial/commentary, abstract with insufficient information, protocol, review article)
Parraga, T. J., McCorquodale, J., Greenlee, S., Osborn, Z., Hanna, Z. W., Williams, J. D., Lanfranco, O. A., Mayur, R., & Alangaden, G. J. (2021). Impact of Pre-Transplant Infectious Diseases Wellness Visit on Vaccine Uptake in Solid Organ Transplant Candidates. *Open Forum Infectious Diseases*, *8*(Supplement_1), S774–S775. https://doi.org/10.1093/ofid/ofab466.1569	Ineligible publication type (e.g., editorial/commentary, abstract with insufficient information, protocol, review article)

Patten, D., Green, A., Bown, D., & Russell, C. (2021). Covid-19: Use social media to maximise vaccine confidence and uptake. *BMJ*, n225. https://doi.org/10.1136/bmj.n225	Ineligible publication type (e.g., editorial/commentary, abstract with insufficient information, protocol, review article)
Poulikakos, D., Chinnadurai, R., Anwar, S., Ahmed, A., Chukwu, C., Moore, J., Hayes, E., Gorton, J., Lewis, D., Donne, R., Lamerton, E., Middleton, R., & O'Riordan, E. (2022). Increasing Uptake of COVID-19 Vaccination and Reducing Health Inequalities in Patients on Renal Replacement Therapy—Experience from a Single Tertiary Centre. *Vaccines*, *10*(6), 939. https://doi.org/10.3390/vaccines10060939	No eligible comparator (i.e., control group)
Ranpariya, V. K., Hrin, M. L., Maghen, P., & Feldman, S. R. (2021). Assessing Behavioral Economic-Based Approaches to Address COVID-19 Vaccine Hesitancy. *Journal of Ambulatory Care Management*, *44*(4), 336–341. https://doi.org/10.1097/jac.0000000000000395	Ineligible publication type (e.g., editorial/commentary, abstract with insufficient information, protocol, review article)
Rosen, A. D., Beltran, J., Thomas, E., Miller, J., Robie, B., Walseth, S., Backes, S., Leachman, N., Chang, A. H., Bratcher, A., Frederes, A., Romero, R., Beas, I., Alvarado, J., Cruz, B., Tabajonda, M., & Shover, C. L. (2022). COVID-19 Vaccine Acceptability and Financial Incentives among Unhoused People in Los Angeles County: a Three-Stage Field Survey. *Journal of Urban Health*, *99*(3), 594–602. https://doi.org/10.1007/s11524-022-00659-x	Ineligible research question (does not evaluate the effectiveness of a non-financial intervention for COVID-19 vaccine hesitancy)
Santos, H. C., Goren, A., Chabris, C. F., & Meyer, M. N. (2021). Effect of Targeted Behavioral Science Messages on COVID-19 Vaccination Registration Among Employees of a Large Health System. *JAMA Network Open*, *4*(7), e2118702. https://doi.org/10.1001/jamanetworkopen.2021.18702	Ineligible outcome (e.g., feelings of vaccine hesitancy, measures of vaccine intentions)
Sehgal, N. K. (2021). Impact of Vax-a-Million Lottery on COVID-19 Vaccination Rates in Ohio. *The American Journal of Medicine*, *134*(11), 1424–1426. https://doi.org/10.1016/j.amjmed.2021.06.032	Ineligible research question (does not evaluate the effectiveness of a non-financial intervention for COVID-19 vaccine hesitancy)
Shekhar, S. K., & Jose, T. P. (2021). Death Anxiety and Mental Health: A Case Analysis of Vaccination Hesitancy and Intervention	Ineligible publication type (e.g.,

Techniques. *Journal of Social Work in End-of-Life & Palliative Care*, *18*(1), 4–7. https://doi.org/10.1080/15524256.2021.2002232	editorial/commentary, abstract with insufficient information, protocol, review article)
Sprengholz, P., Eitze, S., Korn, L., Siegers, R., & Betsch, C. (2021). The power of choice: Experimental evidence that freedom to choose a vaccine against COVID-19 improves willingness to be vaccinated. *European Journal of Internal Medicine*, *87*, 106–108. https://doi.org/10.1016/j.ejim.2021.03.015	Ineligible research question (does not evaluate the effectiveness of a non-financial intervention for COVID-19 vaccine hesitancy)
Su, M., Hughes, B., Testa, M., Goldberg, B., Braddock, K., Miller-Idriss, C., Maturo, V., & Savoia, E. (2021). Testing the Efficacy of Attitudinal Inoculation Videos to Enhance COVID-19 Vaccine Acceptance: A Quasi-Experimental Intervention Trial (Preprint). *JMIR Public Health and Surveillance*. https://doi.org/10.2196/preprints.34116	Ineligible outcome (e.g., feelings of vaccine hesitancy, measures of vaccine intentions)
Syme, M. L., Gouskova, N., & Berry, S. D. (2022). COVID-19 Vaccine Uptake Among Nursing Home Staff via Statewide Policy: The Mississippi Vaccinate or Test Out Policy. *American Journal of Public Health*, *112*(5), 762–765. https://doi.org/10.2105/ajph.2022.306800	Ineligible research question (does not evaluate the effectiveness of a non-financial intervention for COVID-19 vaccine hesitancy)
Takamatsu, A., Honda, H., Kojima, T., Murata, K., & Babcock, H. M. (2021). Promoting coronavirus disease 2019 (COVID-19) vaccination among healthcare personnel: A multifaceted intervention at a tertiary-care center in Japan. *Infection Control & Hospital Epidemiology*, *43*(9), 1201–1206. https://doi.org/10.1017/ice.2021.325	No eligible comparator (i.e., control group)
Talmy, T., Cohen, B., Nitzan, I., & Ben Michael, Y. (2021). Primary Care Interventions to Address COVID-19 Vaccine Hesitancy Among Israel Defense Forces Soldiers. *Journal of Community Health*, *46*(6), 1155–1160. https://doi.org/10.1007/s10900-021-01002-2	No eligible comparator (i.e., control group)
Tarabay, J. (2022). Building Trust to Encourage Vaccination: The Infection Preventionist's Role with Vaccine Compliance. *American Journal of Infection Control*, *50*(7), S28. https://doi.org/10.1016/j.ajic.2022.03.039	Ineligible publication type (e.g., editorial/commentary, abstract with insufficient information, protocol, review article)

The Royal College of Nursing. (2021, May 12). *COVID-19 vaccine refusal: study shows pressure from employers has adverse effect*. https://www.rcn.org.uk/news-and-events/news/uk-covid-19-vaccine-refusal-study-shows-pressure-from-employers-has-adverse-effect-120521	Ineligible publication type (e.g., editorial/commentary, abstract with insufficient information, protocol, review article)
Tuckerman, J., Kaufman, J., & Danchin, M. (2022). Effective Approaches to Combat Vaccine Hesitancy. *Pediatric Infectious Disease Journal*, *41*(5), e243–e245. https://doi.org/10.1097/inf.0000000000003499	Ineligible publication type (e.g., editorial/commentary, abstract with insufficient information, protocol, review article)
Waxman, M. J., Ray, M., Schechter-Perkins, E. M., Faryar, K., Flynn, K. C., Breen, M., Wojcik, S. M., Berry, F., Zheng, A., Ata, A., Lerner, E. B., Lyons, M. S., & McGinnis, S. (2022). Patients' Perspectives on Emergency Department COVID-19 Vaccination and Vaccination Messaging Through Randomized Vignettes. *Public Health Reports*, *137*(4), 774–781. https://doi.org/10.1177/00333549221085580	Ineligible research question (does not evaluate the effectiveness of a non-financial intervention for COVID-19 vaccine hesitancy)
Witus, L. S., & Larson, E. (2022). A randomized controlled trial of a video intervention shows evidence of increasing COVID-19 vaccination intention. *PLOS ONE*, *17*(5), e0267580. https://doi.org/10.1371/journal.pone.0267580	Ineligible outcome (e.g., feelings of vaccine hesitancy, measures of vaccine intentions)
Woon, Y. L., Lee, Y. K., Hyung, J. K., Lothfi, N. M., Wong, E., Perialathan, K., Ahmad Sanusi, N. H., Isa, A., Leong, C. T., & Costa-Font, J. (2022). When do persuasive messages on vaccine safety steer COVID-19 vaccine acceptance and recommendations? Behavioral insights from a randomised controlled experiment in Malaysia. *MedRxiv*. https://doi.org/10.1101/2022.04.17.22273942	Ineligible outcome (e.g., feelings of vaccine hesitancy, measures of vaccine intentions)
Yawn, B., Merrill, D., Martinez, S., Williams, D., Cotton, J., Callen, E., & Loskutova, N. (2021). Comparative vaccine hesitancy in people with COPD. *Chest*, *160*(4), A1850–A1851. https://doi.org/10.1016/j.chest.2021.07.1658	Ineligible research question (does not evaluate the effectiveness of a non-financial intervention for COVID-19 vaccine hesitancy)
Zhu, P., Tatar, O., Griffin-Mathieu, G., Perez, S., Haward, B., Zimet, G., Tunis, M., Dubé, V., & Rosberger, Z. (2022). The Efficacy of a Brief,	Ineligible outcome (e.g., feelings of

Altruism-Eliciting Video Intervention in Enhancing COVID-19 Vaccination Intentions Among a Population-Based Sample of Younger Adults: Randomized Controlled Trial. *JMIR Public Health and Surveillance*, *8*(5), e37328. https://doi.org/10.2196/37328	vaccine hesitancy, measures of vaccine intentions)

Table S9. Risk of bias assessments for randomized intervention studies included in review*

Citation	Domain	Risk of bias judgement	Comments
Dai et al., 2021 [1]	Overall	Low	Low risk assigned for all 5 domains
	1. Risk of bias arising from the randomization process	Low	No concerns regarding randomization, concealment, or differences in baseline characteristics between groups
	2. Risk of bias due to deviations from the intended interventions	Low	No deviations notes
	3. Missing outcome data	Low	No concerns relating to missing outcome data
	4. Risk of bias in measurement of the outcome	Low	No evidence of bias relating to outcome measurement
	5. Risk of bias in selection of the reported result	Low	Reported result in accordance with pre-specified analysis plan
Lieu et al., 2022 [2]	Overall	Some concerns	Overall concerns relating to randomization and outcome measurement
	1. Risk of bias arising from the randomization process	Some concerns	Allocation sequence concealment not specified in either the manuscript or protocol
	2. Risk of bias due to deviations from the intended interventions	Low	No deviations noted
	3. Missing outcome data	Low	No concerns relating to missing outcome data
	4. Risk of bias in measurement of the outcome	Some concerns	Not specified in the manuscript or protocol whether

			outcome assessors aware of the intervention received by study participants
	5. Risk of bias in selection of the reported result	Low	Reported result in accordance with pre-specified analysis plan
Mehta et al., 2022 [3]	Overall	Low	Low risk assigned for all 5 domains
	1. Risk of bias arising from the randomization process	Low	No concerns regarding randomization, concealment, or differences in baseline characteristics between groups
	2. Risk of bias due to deviations from the intended interventions	Low	No deviations noted
	3. Missing outcome data	Low	No concerns relating to missing outcome data
	4. Risk of bias in measurement of the outcome	Low	No evidence of bias relating to outcome measurement
	5. Risk of bias in selection of the reported result	Low	Reported result in accordance with pre-specified analysis plan
Tentori et al., 2022 [4]	Overall	Low	Possible concern with missing outcome data but otherwise low risk assigned in other 4 domains
	1. Risk of bias arising from the randomization process	Low	No concerns regarding randomization, concealment, or differences in baseline characteristics between groups

	2. Risk of bias due to deviations from the intended interventions	Low	No deviations noted
	3. Missing outcome data	Some concerns	No information provided in the manuscript regarding missing outcome data
	4. Risk of bias in measurement of the outcome	Low	No evidence of bias relating to outcome measurement
	5. Risk of bias in selection of the reported result	Low	Reported result in accordance with pre-specified analysis plan

***Cochrane Risk of Bias tool (RoB 2) Domain Descriptions**

A ‘low’ risk of bias judgement in the randomization process domain is characterized by a random allocation sequence, allocation concealment, and assessment of baseline characteristics between study arms/groups.

A ‘low’ risk of bias judgement in the deviations from intended interventions domain is characterized by blinding of participants and carers/researchers and an appropriate analysis to estimate the effect of assignment to intervention.

A ‘low’ risk of bias judgement in the missing outcome data domain is characterized by outcome data being for all (or nearly all) participants that were randomized and if there was missing outcome data, no evidence to suggest that the result was biased by missing outcome data.

A ‘low’ risk of bias judgement in the outcome measurement domain is characterized by an appropriate method of measuring the outcome, no difference in the method of measurement/ascertainment of the outcome between groups, and the outcome assessors being blinded to the intervention received by participants.

A ‘low’ risk of bias judgement in the selection of reported result domain is characterized by following a pre-specified analysis plan and a numerical result that was likely not selected from multiple eligible outcome measurements or multiple eligible analyses of the outcome data.

Table S10. Risk of bias assessments for non-randomized intervention studies included in review**

Citation	Signalling questions category	Risk of bias judgement	Comments
Chan et al., 2022 [5]	Overall	Moderate	Overall concerns relating to confounding and measurement of outcomes
	1. Bias due to confounding	Moderate	No mention of controlling for confounders by the authors
	2. Bias in selection of participants into the study	Low	No evidence of selection bias
	3. Bias in classification of interventions	Low	No evidence of bias relating to intervention group classification
	4. Bias due to deviations from intended interventions	Low	No deviations noted
	5. Bias due to missing data	Low	No evidence of bias relating to missing data
	6. Bias in measurement of outcomes	Moderate	Outcome assessors were aware of the intervention received by participants; however, the outcome measure was likely not influenced by knowledge of intervention received
	7. Bias in selection of the reported result	Low	No evidence of the reported result being selected from multiple sub-group and/or intervention-outcome relationship analyses
Crutcher & Seidler, 2021[6]	Overall	Moderate	Overall concerns relating to confounding and

			measurement of outcomes
	1. Bias due to confounding	Serious	No mention of controlling for confounder by the authors, and no individual demographic data collected from participants to contextualize study findings
	2. Bias in selection of participants into the study	Low	No evidence of selection bias
	3. Bias in classification of interventions	Low	No evidence of bias relating to intervention group classification
	4. Bias due to deviations from intended interventions	Low	No deviations noted
	5. Bias due to missing data	Low	No evidence of bias relating to missing data
	6. Bias in measurement of outcomes	Moderate	Outcome assessors were aware of the intervention received by participants; however, the outcome measure was likely not influenced by knowledge of intervention received
	7. Bias in selection of the reported result	Low	No evidence of the reported result being selected from multiple sub-group and/or intervention-outcome relationship analyses

****Cochrane Risk of Bias in Non-randomized Studies - of Interventions (ROBINS-I) Domain Descriptions**

A 'low' risk of bias judgement in the confounding domain is characterized by no potential for confounding of the effect of the intervention, or if there is potential for confounding, appropriate assessment of and controlling for temporal and baseline confounding factors.

A 'low' risk of bias judgement in the selection of participants domain is characterized by selection of participants not being based on participant characteristics observed after the start of intervention and follow-up/start of intervention coinciding for most participants.

A 'low' risk of bias judgement in the classification of interventions domain is characterized by clearly defined intervention groups, information used to define intervention groups being recorded at the start of the intervention, and classification of intervention status not being affected by knowledge of the outcome/risk of the outcome.

A 'low' risk of bias judgement in the deviations from intended interventions domain is characterized by no deviations from the intended intervention beyond what would be expected in usual practice, and if there were deviations, they were well balanced between groups and unlikely to affect the outcome.

A 'low' risk of bias judgement in the missing data domain is characterized by outcome data being available for all (or nearly all) participants and no exclusion of participants to missing data on intervention status or other variables needed for the analysis.

A 'low' risk of bias judgement in the outcome measurement domain is characterized by an outcome measurement that was likely not influenced by knowledge of intervention received, comparable outcome assessment across groups, blinding outcome assessors to the intervention received by the participant, and no systematic error in the outcome measurement related to intervention received.

A 'low' risk of bias judgement in the selection of reported result domain is characterized by a reported effect estimate that likely not selected on the basis of results from multiple outcome measurements, multiple analyses of the intervention-outcome relationship, or different sub-groups.

References

1 Dai, H., Saccardo, S., Han, M.A., Roh, L., Raja, N., Vangala, S., Modi, H., Pandya, S., Sloyan, M., and Croymans, D.M.: 'Behavioural nudges increase COVID-19 vaccinations', Nature, 2021, 597, (7876), pp. 404-409

2 Lieu, T.A., Elkin, E.P., Escobar, P.R., Finn, L., Klein, N.P., Durojaiye, C., Prausnitz, S., Quesenberry, C.P., Sawyer, D., Teran, S., Goler, N., Parodi, S.M., and Chen, Y.-F.I.: 'Effect of Electronic and Mail Outreach From Primary Care Physicians for COVID-19 Vaccination of Black and Latino Older Adults: A Randomized Clinical Trial', JAMA Netw Open, 2022, 5, (6), pp. e2217004-e2217004

3 Mehta, S.J., Mallozzi, C., Shaw, P.A., Reitz, C., McDonald, C., Vandertuyn, M., Balachandran, M., Kopinsky, M., Sevinc, C., Johnson, A., Ward, R., Park, S.-H., Snider, C.K., Rosin, R., and Asch, D.A.: 'Effect of Text Messaging and Behavioral Interventions on COVID-19 Vaccination Uptake: A Randomized Clinical Trial', JAMA Netw Open, 2022, 5, (6), pp. e2216649-e2216649

4 Tentori, K., Pighin, S., Giovanazzi, G., Grignolio, A., Timberlake, B., and Ferro, A.: 'Nudging COVID-19 Vaccine Uptake by Changing the Default: A Randomized Controlled Trial', Medical Decision Making, 2022, 42, (6), pp. 837-841

5 Chan, D.K., Alegria, B.D., Chadaga, S.R., Goren, L.J., Mikasa, T.J., Pearson, A.M., Podolsky, S.R., Won, R.S., and LeTourneau, J.L.: 'Rapid Deployment of Multiple Tactics to Address Severe Acute Respiratory Syndrome Coronavirus 2 Vaccine Uptake in Healthcare Employees With a Focus on Those Who Identify as Black, Indigenous, and People of Color', Open Forum Infect Dis, 2022, 9, (3), pp. ofac012-ofac012

6 Crutcher, M., and Seidler, P.M.: 'Maximizing Completion of the Two-Dose COVID-19 Vaccine Series with Aid from Infographics', Vaccines (Basel), 2021, 9, (11), pp. 1229
